# Medical Marijuana and Treatment Personalization: The Role of Genetics and Epigenetics in Response to THC and CBD

**DOI:** 10.3390/genes16121487

**Published:** 2025-12-12

**Authors:** Małgorzata Kalak, Anna Brylak-Błaszków, Łukasz Błaszków, Tomasz Kalak

**Affiliations:** 1 Faculty of Medicine, Prince Mieszko I Poznan Medical University of Applied Sciences, Bułgarska 55, 60-320 Poznań, Poland; 2 GenXone SA, Kobaltowa 6, 62-002 Złotniki, Poland; 3 Independent Researcher, 60-542 Poznań, Poland; 4 Department of Industrial Products and Packaging Quality, Institute of Quality Science, Poznań University of Economics and Business, Niepodległości 10, 61-875 Poznań, Poland

**Keywords:** medical marijuana, tetrahydrocannabinol (THC), cannabidiol (CBD), pharmacogenomics, drug–drug interactions (DDIs), phenoconversion, epigenetics

## Abstract

Personalizing therapy using medical marijuana (MM) is based on understanding the pharmacogenomics (PGx) and drug–drug interactions (DDIs) involved, as well as identifying potential epigenetic risk markers. In this work, the evidence regarding the role of variants in phase I (*CYP2C9*, *CYP2C19*, *CYP3A4/5*) and II (*UGT1A9*/*UGT2B7*) genes, transporters (*ABCB1*), and selected neurobiological factors (*AKT1*/*COMT*) in differentiating responses to Δ^9^-tetrahydrocannabinol (THC) and cannabidiol (CBD) has been reviewed. Data indicating enzyme inhibition by CBD and the possibility of phenoconversion were also considered, which highlights the importance of a dynamic interpretation of PGx in the context of current pharmacotherapy. Simultaneously, the results of epigenetic studies (DNA methylation, histone modifications, and ncRNA) in various tissues and developmental windows were summarized, including the reversibility of some signatures in sperm after a period of abstinence and the persistence of imprints in blood. Based on this, practical frameworks for personalization are proposed: the integration of PGx testing, DDI monitoring, and phenotype correction into clinical decision support systems (CDS), supplemented by cautious dose titration and safety monitoring. The culmination is a proposal of tables and diagrams that organize the most important PGx–DDI–epigenetics relationships and facilitate the elimination of content repetition in the text. The paper identifies areas of implementation maturity (e.g., *CYP2C9*/THC, CBD-*CYP2C19*/clobazam, *AKT1,* and acute psychotomimetic effects) and those requiring replication (e.g., multigenic analgesic signals), indicating directions for future research.

## 1. Introduction

The use of medical marijuana (MM) preparations, primarily Δ^9^-tetrahydrocannabinol (THC) and cannabidiol (CBD), is rapidly increasing across various clinical indications, while there is significant inter-individual variability in efficacy and safety. The biological basis for this heterogeneity involves the complex signaling of the endocannabinoid system (ECS) and pharmacokinetic–pharmacodynamic (PK/PD) variability resulting from drug metabolism and interactions [1]. At the level of clinical practice, understanding when and how to incorporate genetic or epigenetic data into the selection of the preparation, route of administration, and dose titration remains crucial to maximize benefits and minimize risks.

From a pharmacogenomics (PGx) perspective, the metabolism of THC and CBD is primarily determined by oxidative phase I pathways involving CYP2C9, CYP2C19, and CYP3A4/5 and phase II glucuronidation involving UGT1A9/UGT2B7 [2,3,4,5,6]. Biochemical and translational data indicate that the *2/*3 variants in *CYP2C9* slow down THC clearance and increase exposure (AUC), which is relevant for the risk of central effects after oral administration [7]. In the case of CBD, the key 7-hydroxylation occurs with the involvement of CYP2C19 [4], and CBD inhibits CYP2C19/3A, which creates a basis for clinically significant DDIs—classically with clobazam and the accumulation of *N*-desmethylclobazam [5,6,8]. Additionally, UGT inhibitors (CBD > THC) can modulate the glucuronidation of drugs with a narrow therapeutic window [6]. The involvement of transporters (e.g., *ABCB1*) and ECS components (*CNR1*/*CNR2* and *FAAH*), as well as neurobiological modifiers (*AKT1*/*COMT*), completes the picture of potential sources of response variability [1,9,10,11].

Simultaneously, epigenetic research is growing: EWAS studies and experimental research link cannabis use with DNA methylation alterations in peripheral blood, exposure markers, and accelerated epigenetic age in selected contexts [12]. Controlled exposures show a concentration-dependent effect of THC on DNMT/TET expression, and in males, some methylation changes in sperm are reduced after ≈1 spermatogenesis cycle, suggesting a reversible component; however, persistent signatures remain in the blood, which is a major point of debate regarding the extent of reversibility and clinical significance [13,14]. During sensitive periods (pregnancy and adolescence), methylation signals with potential developmental consequences are observed, but they require rigorous co-exposure control (tobacco, alcohol, and pharmacotherapy) before any meaningful conclusions can be drawn [15].

The status of clinical research remains diverse across indications. For chronic pain, systematic reviews and BMJ guidelines indicate small-to-moderate analgesic effects and the need to balance benefits against risks [16,17], while more recent RCTs for a specific, standardized extract suggest a clinically significant reduction in pain intensity, with limited generalizability to other formulations [18]. For spasticity in MS, the evidence is moderate (including for a THC/CBD oromucosal spray) [19], while for developmental epilepsies, high-quality RCTs have established the efficacy of purified CBD and created a personalization standard involving close monitoring of interactions (clobazam and valproic acid) [8,20]. Controversies also persist regarding the “entourage effect” hypothesis, which has numerous pharmacological bases but limited clinical validation; caution is advised in extrapolations and preference should be given to preparations with a verified composition [21]. In neuropsychiatry, the importance of *AKT1* (acute psychotomimetic effects of THC) is growing, while the *COMT* × cannabis interaction was not confirmed in a meta-analysis, which limits the usefulness of routine *COMT* testing [10,11].

The purpose of this work is twofold. Firstly, the evidence regarding the role of genetics (*CYP2C9*, *CYP2C19*, *UGT2B7*, *ABCB1,* and *AKT1*/*COMT*) and epigenetics in response to THC/CBD and safety of use in polytherapy is systematically reviewed and organized based on the literature. Secondly, this evidence has been translated into a personalization framework encompassing: (i) integration of PGx with DDI logic and dynamic phenotype correction (phenoconversion) in clinical decision support systems, (ii) selection of the route of administration and formulation composition based on the patient profile, and (iii) careful titration and safety monitoring [22,23].

## 2. Materials and Methods

### 2.1. Research

The work is a narrative review with elements of a methodological overview of implementation, focusing on PGx, DDIs, and epigenetics in MM personalization. The aim was to synthesize and organize evidence regarding the personalization of MM (THC/CBD) therapy in the context of pharmacogenomics (PGx), drug–drug interactions (DDIs), and epigenetics.

### 2.2. Information Sources and Search Strategy

Systematic searches were conducted in the MEDLINE (PubMed), Embase, Scopus, ScienceDirect, and Cochrane Library databases, supplemented by a review of the bibliographies of key works. The publications sought had no restrictions regarding country and were published in English in various journals from the JCR list. The example query included combinations of keywords such as MM, cannabis, THC, Δ^9^-tetrahydrocannabinol, cannabidiol, CBD, pharmacogen, *CYP2C9*, *CYP2C19*, *CYP3A*, *UGT*, *ABCB1*, *AKT1*, *COMT*, epigen, methylation, or epigenome-wide.

Articles published in the literature were searched regarding the following: (i) THC/CBD pharmacogenetics; (ii) drug–gene interactions related to MM; (iii) the modifying role of genes in the neuropsychiatric and analgesic effects of cannabinoids. Priority was given to human studies (healthy volunteers and patients), meta-analyses, and studies with clearly defined clinical or pharmacokinetic endpoints.

The following study types were included: (i) human studies (RCTs, pragmatic studies, cohort studies, case–control studies, cross-sectional studies, and N-of-1 studies); (ii) pharmacokinetic/pharmacodynamic (PK/PD) studies on THC/CBD; (iii) PGx studies involving at least one of the genes *CYP2C9*, *CYP2C19*, *CYP3A4/5*, *UGT1A9/UGT2B7*, *ABCB1*, *AKT1*, or *COMT*; (iv) epigenetic studies (including EWAS) on cannabis/THC/CBD exposure; (v) systematic reviews and meta-analyses synthesizing clinical or mechanistic data.

## 3. Fundamentals of Genetics and Epigenetics in the Context of Marijuana

**Figure 1:**
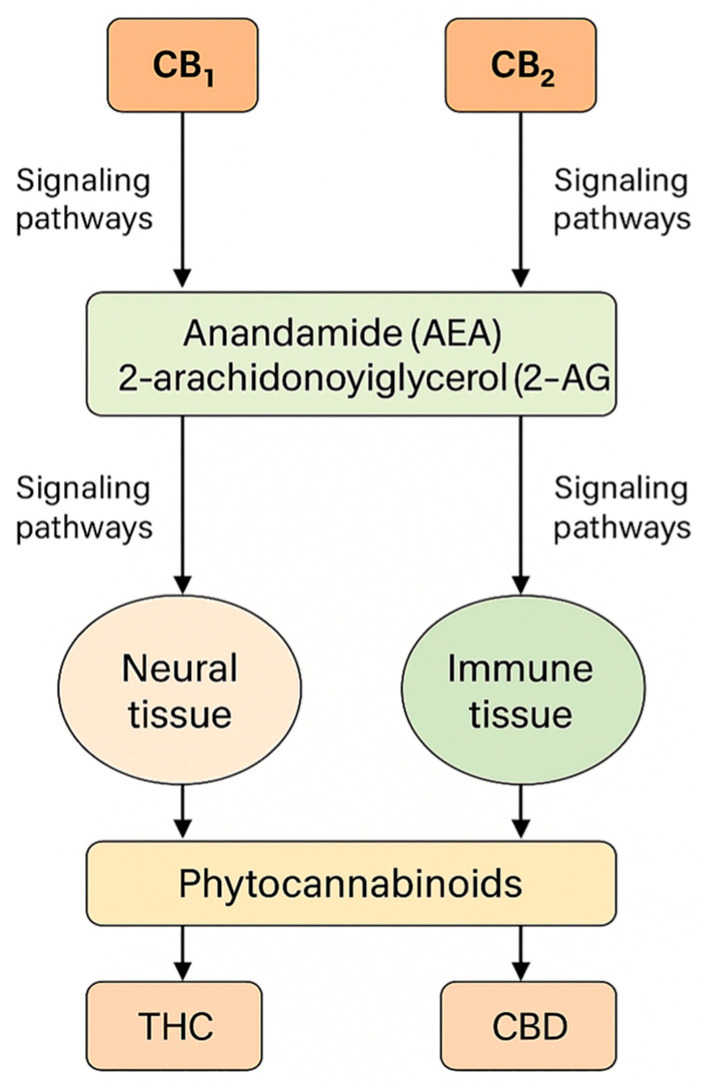
The endocannabinoid system: structure, receptors, and signaling pathways.

The widespread distribution of the endocannabinoid system (ECS) in the central and peripheral nervous systems, as well as in immune and metabolic tissues, provides a biological basis for the action of phytocannabinoids, particularly Δ^9^-tetrahydrocannabinol (THC) and cannabidiol (CBD). THC is a molecule with the characteristics of a partial agonist of CB_1_/CB_2_ receptors, which—along with the signaling properties of the *GPCR* receptors themselves—significantly determines the psychoactive and somatic profile; recent structural–functional analyses of CB_1_/CB_2_ and ligand–receptor interaction modeling are deepening our understanding of this partial agonism and individual response variability [1]. To provide a visual overview of the endocannabinoid system and its main molecular components, Figure 1 summarizes receptor distribution, endogenous ligands, and major signaling pathways relevant to cannabinoid activity.

CBD acts through multiple mechanisms (including interactions with 5-HT1A, TRP receptors, and ion channels), which explains the range of observed pharmacological effects depending on dose, route of administration, and patient physiology. In this chapter, the basics of genetics and epigenetics related to cannabis were synthesized, from the influence of genetic variants on the pharmacokinetics/pharmacodynamics of THC/CBD to epigenetic modifications accompanying acute, chronic, prenatal, or periconceptional exposure. At the same time, it should be emphasized that the personalization of cannabinoid therapy, using pharmacogenomic data and epigenetic biomarkers, is clinically relevant given the significant inter-individual variability in efficacy [1].

**Figure 2:**
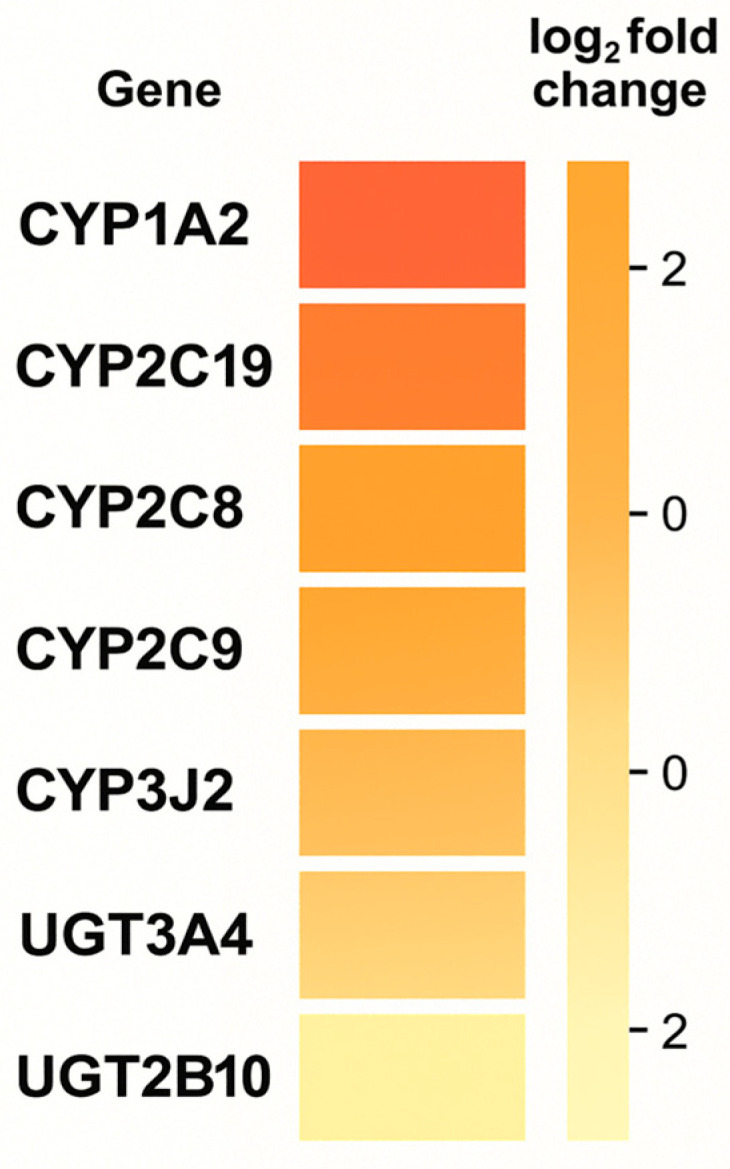
Heatmap of differential gene expression (log_2_ fold change) associated with cannabinoid exposure.

From a pharmacogenomics perspective, variability in genes encoding biotransformation enzymes (e.g., *CYP2C9*, *CYP2C19*, *CYP3A*) and in elements of the endocannabinoid pathway can modulate the active metabolite concentration, duration of action, and range of adverse effects. While some conclusions are based on generating hypotheses (a limited number of studies with hard clinical endpoints), the available reviews and experimental studies indicate that considering the genetic profile can improve response predictability and reduce the risk of drug interactions, justifying the development of “PGx-aware” cannabinoid medicine [2]. As illustrated in Figure 2, the differential expression pattern of genes across experimental conditions shows a clear distinction between control and treated samples, with a predominance of upregulation within the *CYP* gene family and moderate downregulation within the *UGT* cluster. Figure 2 provides a schematic illustration of differential expression patterns of selected *CYP* and *UGT* genes observed across cannabinoid-exposed vs. control conditions in experimental datasets; it is not intended to imply that CYP1A2 is the primary driver of cannabinoid PK. The subsequent PGx discussion (Section 3.1.1) is based on enzyme-specific PK and clinical data, which support CYP2C9 and CYP2C19 as key determinants of THC and CBD clearance, respectively.

Epigenetics brings a distinct, complementary dimension: exposure to cannabinoids is associated with DNA methylation modifications and changes in gene expression in various tissues which are also dependent on life stage. In adult users, epigenetic traces of cannabis smoking have been described (also persisting after exposure); in men, methylation changes in sperm with partial reversibility after ~11 weeks of abstinence (one spermatogenesis cycle); and in studies on prenatal exposure, methylation signatures in offspring involving genes related to nervous system development. These observations highlight the potential reversibility of some effects and the importance of so-called “sensitive windows”, while also requiring rigorous control of confounding factors (e.g., tobacco, alcohol) and replication in large cohorts [12].

Pharmacogenomics (PGx) is a field that studies the relationship between DNA variants and drug response—both pharmacokinetics (absorption, metabolism, and clearance) and pharmacodynamics (receptor sensitivity and signaling pathways)—with the aim of optimizing the effectiveness and safety of therapy at the individual level. Epigenetics refers to mitotically and/or meiotically heritable changes in gene function that do not result from changes in DNA sequence; it includes, among other things, DNA methylation, histone modifications, and the actions of non-coding RNAs. Such changes can record traces of environmental exposures, including those of cannabinoids. The key practical difference is that the effects of acute exposure (e.g., temporary modulation of brain network connectivity after THC) primarily concern transient physiological states, whereas persistent epigenetic changes can—at least partially—persist after exposure ends and potentially influence phenotype or offspring development [24].

Consequently, this chapter focuses on (i) mapping the main pharmacological mechanisms of THC/CBD within the ECS, (ii) reviewing genetic variants with the greatest clinical potential in modulating cannabinoid response, and (iii) synthesizing evidence for the epigenetic effects of exposure—distinguishing between acute effects and sustained changes—to identify areas where therapy personalization is already feasible and areas requiring further validation [1].

### 3.1. Pharmacogenomics of Cannabinoids (THC, CBD, and Other Phytocannabinoids)

#### 3.1.1. Phase I Enzymes: *CYP2C9*, *CYP2C19*, *CYP3A4/5*

Phase I enzymes play a rather well-characterized key role in the biotransformation of phytocannabinoids. For Δ^9^-tetrahydrocannabinol (THC), the main oxidation pathway is CYP2C9: reduced-activity variants (*2, *3) slow down clearance and may increase systemic exposure and the risk of dose-dependent effects; CYP3A isoforms (mainly 3A4/5) and CYP2C19 also contribute to THC clearance, albeit to a lesser but measurable extent, as confirmed by studies using liver microsomes, recombinant enzymes, and more recent metabolomic analyses of THC and its oxidation products [3]. In the biotransformation of cannabidiol (CBD), 7-hydroxylation of CBD to its active metabolite 7-OH-CBD is primarily catalyzed by *CYP2C19*; data from human liver microsomes genotyped for *CYP2C19* indicate that polymorphisms of this gene modulate the rate of 7-OH-CBD formation, and CYP3A4/5 as well as CYP2C9—although to a lesser extent—contribute to other oxidative pathways of CBD [4]. Additionally, CBD has been shown in vitro to inhibit several CYP isoforms, particularly CYP3A, CYP1A2, and CYP2C19, including time-dependent inhibition, which provides a mechanistic rationale for the observed potential for drug interactions in PBPK modeling and pharmacokinetic reviews [5].

In light of recent research, the role of *CYP2C9* in THC clearance has been well-established: carrying alleles that reduce activity (*2, *3) is associated with slower elimination resulting in higher exposure to THC/11-OH-THC, which can exacerbate its effects; the contribution of CYP3A4/5 and CYP2C19 to THC oxidation is smaller but measurable (including 8-hydroxylation by CYP3A4). In contrast, the formation of 7-OH-CBD by CYP2C19 is crucial in CBD biotransformation; loss-of-function polymorphisms (*2, *3) decrease the rate of this pathway, while *17 (GOF) can increase it. In terms of drug interactions, CBD inhibits several CYP isoforms in vitro (depending on the time, including CYP3A and CYP2C19), although the magnitude of the in vivo effects is often smaller than predicted based on in vitro data/PBPK models. A summary of the key gene–metabolism phenotype relationships for THC/CBD is presented in Table 1. Further clinical implications (dosage and safety) are discussed in Section 4.1 and Section 4.2.

Data in Table 1 are derived from human PK studies and mechanistic in vitro work where available. For pathways with no direct clinical outcome data (e.g., *CYP3A5*, some *CYP3A4* variants), predictions remain hypothesis-generating and are marked as “limited” or “unclear”.

**Figure 3:**
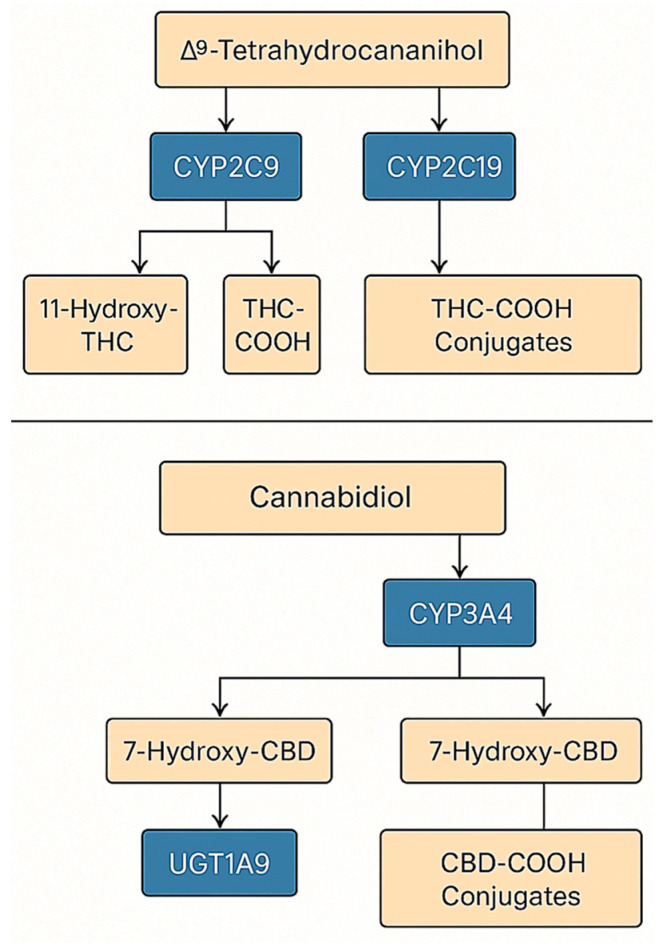
Integrated model of THC and CBD metabolism.

To provide a comprehensive view of the enzymatic conversion of cannabinoids, Figure 3 summarizes the integrated metabolic network of THC and CBD, combining both oxidative (CYP-mediated) and conjugative (UGT-mediated) phases. This model highlights major pathways responsible for metabolite formation and potential drug–drug interaction sites relevant to pharmacokinetic variability.

#### 3.1.2. Phase II Enzymes: UGT

**Figure 4:**
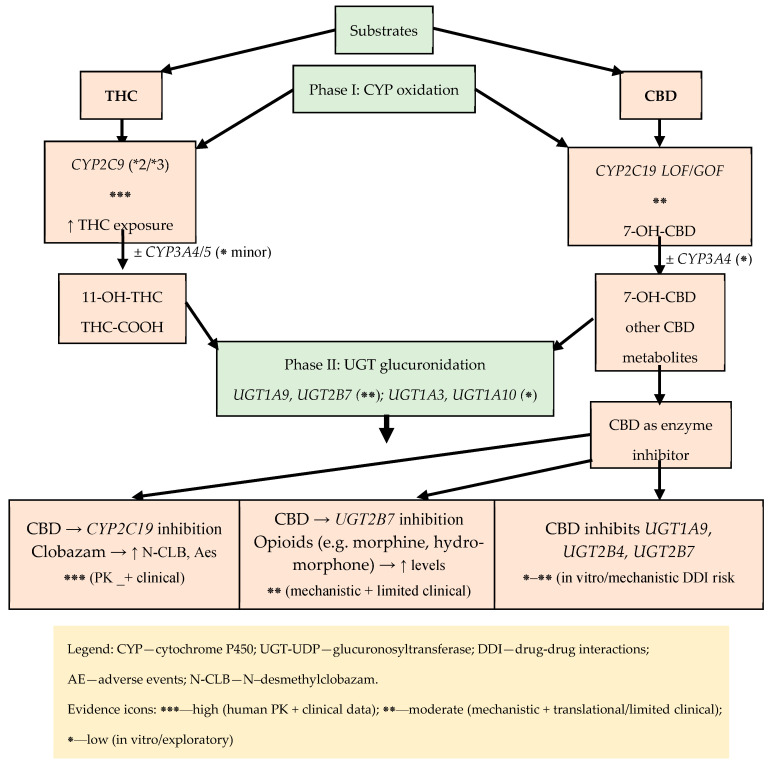
Metabolic map of THC/CBD (phase I: CYP; phase II: UGT) with key DDI nodes and strength of evidence.

In the context of phase II metabolism, the glucuronidation of oxidative THC metabolites—particularly 11-OH-THC and 11-nor-9-carboxy-THC (THC-COOH)—is critical, involving several UGT isoenzymes, primarily UGT1A9, as well as UGT1A3, UGT1A10, and UGT2B7; although this process is well-documented biochemically, its clinical implications remain more elusive than those of phase I reactions. Simultaneously, exposure to cannabinoids can modulate this pathway: in vitro studies demonstrate that CBD is a strong inhibitor of UGT1A9, UGT2B4, and UGT2B7 activity (THC inhibits them less strongly), which creates a plausible mechanism for pharmacokinetic drug–drug interactions for substrates of these enzymes; predictions and experimental data draw particular attention to the possibility of impaired glucuronidation of opioids such as morphine (UGT2B7) or hydromorphone, which could potentially alter exposure to active metabolites (M6G) and clinical as well as adverse effects [6]. Several functional polymorphisms have been described in *UGT1A9* and *UGT2B7*, but to date there are no robust data linking specific UGT genotypes to THC or CBD exposure or clinical outcomes. Consequently, our discussion of UGTs focuses on their role as DDI nodes (inhibition by CBD and, to a lesser extent, THC) rather than on a defined UGT pharmacogenomic framework. Figure 4 presents an integrated, two-step metabolic map of THC and CBD (phase I and phase II) that also highlights key DDI nodes and the strength of evidence for each pathway. In phase I, oxidative transformations of THC catalyzed by CYP2C9 are dominant (influence of functional variants *2/3 on clearance), with a smaller contribution from *CYP3A4/5* (e.g., CYP3A5*3) and CYP2C19 (LOF *2, GOF *17), leading to 11-OH-THC (to THC-COOH) and CBD metabolites (e.g., 7-OH-CBD). In phase II, there is glucuronidation by UGT1A9, UGT1A3, UGT1A10, and UGT2B7.

#### 3.1.3. *CNR1*/*CNR2* Biology and Current Limits of Receptor Pharmacogenomics

The endocannabinoid system (ECS) comprises the cannabinoid receptors CB1 and CB2, their endogenous ligands anandamide (AEA) and 2-arachidonoylglycerol (2-AG), and the enzymes responsible for endocannabinoid synthesis and degradation [25]. CB1 is encoded by *CNR1* on chromosome 6 (q15), which contains a single protein-coding exon and alternative 5′UTRs and N-terminal variants (CB1a/CB1b) that modulate receptor pharmacodynamics without altering the core protein sequence [26]. CB2 is encoded by *CNR2* on chromosome 1 (1p36.11) and consists of two exons; transcriptional variants (CB2A/CB2B) differ in the 5′UTR but share an identical protein product [27]. CB1 is highly expressed in the central nervous system, particularly at presynaptic terminals, where it tonically regulates GABA and glutamate release and thereby synaptic plasticity [28], whereas CB2 is predominant in immune cells and microglia, with low baseline expression in the brain that increases in neuroinflammatory states [29]. Endocannabinoids are produced “on demand” (AEA via NAPE-PLD; 2-AG via DAGL) and degraded mainly by FAAH (AEA) and MAGL (2-AG); 2-AG is a full CB1/CB2 agonist, while AEA is a partial agonist, leading to distinct signaling dynamics [30].

CB1 and CB2 belong to the GPCR superfamily and primarily couple to Gi/o, inhibiting adenylyl cyclase and cAMP production and engaging MAPK (ERK1/2, JNK, and p38) and PI3K/Akt cascades [31]. CB1 regulates presynaptic ion channels by inhibiting N-, P/Q-, and R-type Ca^2+^ channels and activating GIRK channels, thereby suppressing vesicular exocytosis and neurotransmitter release [32]. Depending on the ligand and the cellular context, CB1 can also signal via Gs or Gq/11, contributing to functional selectivity (biased signaling) [31]. After phosphorylation by GRKs, CB1 and CB2 recruit β-arrestins, leading to desensitization, receptor internalization, and alternative signaling; structural and functional studies demonstrate biased agonism at both receptors and suggest that different ligands or allelic variants can favor distinct β-arrestin recruitment profiles [33,34,35]. CB1/CB2 also form homo- and heteromers with other GPCRs (e.g., CB1–A2A, CB1–D2, CB1–OX1, CB1–CB2, and CB2–GPR55), which modifies pharmacology and downstream signaling [36]. Recent cryo-EM and X-ray structures of CB1/CB2 in complex with Gi and ligands clarify the conformational basis of transducer selection and biased signaling, including a role for ICL2 and membrane lipids such as cholesterol [37].

At the systems level, CB1 and CB2 integrate neuronal and immune signals across multiple organs. CB1 in the liver, adipose tissue, and pancreas contributes to metabolic regulation (e.g., lipogenesis, cAMP signaling) and helps to explain both the adverse effects and therapeutic potential of peripherally selective CB1 ligands, while CB2, which lacks psychoactive effects, remains an attractive target in inflammatory and neuropathic pain and immune-mediated disorders, including neuroinflammatory conditions [38].

##### PGx Relevance

Several common *CNR1* and *CNR2* polymorphisms (e.g., *CNR1 rs1049353, CNR2 rs35761398/Q63R*) have been associated with neuropsychiatric, metabolic, and immune phenotypes; however, the findings are heterogeneous, effect sizes appear small or context-dependent, and replications are often negative [39]. To date, no consistent, clinically actionable associations between *CNR1/CNR2* genotype and medical cannabis response have been demonstrated. Therefore, we do not currently recommend routine *CNR1/CNR2* testing for MM personalization.

#### 3.1.4. Endocannabinoid Degradation Enzymes: *FAAH* (C385A/rs324420)

Fatty acid amide hydrolase (FAAH) is a key hydrolase that terminates endocannabinoid signaling by breaking down substances like anandamide (AEA). The functional polymorphism rs324420 (c.385C>A; p.Pro129Thr) reduces the stability of the FAAH protein, increases AEA levels, and modulates the excitability of limbic pathways, resulting in differential phenotypic susceptibility (including to substance use disorders, obesity, and anxiety reactivity). The literature data includes biochemical studies, human imaging, translational work on a knock-in model, and meta-analyses [40].

*FAAH* catalyzes the hydrolysis of fatty acid amides (AEAs and related N-acylethanolamides), limiting the duration and range of the endocannabinoid signal. The relationship between FAAH levels and amygdala function has been demonstrated through molecular and functional imaging in humans: lower FAAH concentrations are associated with a dampened amygdala response to threatening stimuli [41].

The 385C>A change results in a Pro129Thr substitution in the FAAH catalytic domain, making the protein more susceptible to proteolytic degradation. In homozygous A/A cells, the concentration and activity of FAAH are significantly reduced due to post-transcriptional and post-translational instability; this effect was also confirmed in expression systems [40]. Early reports linked the variant to the use of psychoactive substances in population association studies [42].

Reduced FAAH activity/stability in carriers of the A allele increases AEA levels and shifts the signaling balance toward *CB1* receptor agonism, which manifests as changes in fronto-amygdala connectivity and reduced amygdala reactivity. These data come from (i) human fMRI studies, (ii) PET studies (linking enzyme levels to amygdala reactivity), and (iii) translational work with a C385A knock-in mouse, demonstrating the convergence of neuronal and behavioral phenotypes [43]. The A allele modulates the mechanisms of fear extinction and extinction memory, which has been confirmed in human fear conditioning paradigms (fMRI) [44].

Historical association studies have linked rs324420 to substance-abuse phenotypes, and more recent data suggest that lower FAAH activity promotes increased alcohol consumption (including binge episodes) and riskier drinking patterns in young adults; however, not all studies replicate the associations with nicotine. Concurrently, reduced levels of FAAH were found in the brains of individuals seeking treatment for alcohol addiction in PET imaging. Together, this creates a cohesive, albeit heterogeneous, picture of the FAAH–AEA axis-dependent risk and symptom severity [40].

Early case–control studies linked the homozygous A/A variant to overweight and obesity, and a 2023 systematic review (28 studies, 28,183 participants) indicated “some evidence” of higher BMI and fat mass, as well as changes in glucose–lipid metabolism in carriers of the variant allele—with significant heterogeneity in results and context-dependent effects [45].

Reports link rs324420 to anxiety traits (based on the fronto-amygdaloid mechanisms described above) and—in case studies and functional genetics—to analgesia modulation within the *FAAH/FAAH-OUT* locus (with *FAAH-OUT* microdeletion causing extremely low FAAH activity and an analgesia phenotype; this does not directly involve rs324420, but it strengthens the biological plausibility of the FAAH–AEA axis for pain). Preliminary observations of an association with generalized epilepsy have also emerged [46].

The frequency of allele A varies between populations, and the magnitude of the phenotypic effect depends on the environment and study design. However, translational studies (from cells, to a knock-in model, to human imaging) consistently indicate that rs324420 is a variant that reduces FAAH stability/levels and increases *AEA* tone, even if the translation to clinical risk is variable [40].

Due to the influence of rs324420 on FAAH activity and endocannabinoid tone, this polymorphism is considered a potential modifier of the response to pharmacological interventions targeting the ECS (e.g., FAAH inhibitors) and as a risk/stratification marker in studies on AUD and anxiety. Imaging and behavioral data suggest that reducing FAAH activity may enhance the positive effects of alcohol and the motivation to drink, which justifies the careful design of genotype-informed clinical trials [47].

#### 3.1.5. Dopaminergic Genes/Modulators: *COMT Val158Met*

Catechol-O-methyltransferase (COMT) is a key enzyme in the degradation of catecholamines (including dopamine) in the central nervous system, with a particularly important role in the prefrontal cortex (PFC), where low dopamine transporter expression makes enzymatic degradation a major pathway for signal termination, in contrast to the striatum [48]. The *COMT* gene encodes soluble (*S-COMT*) and membrane-bound (MB-*COMT*) isoforms; the common non-synonymous Val158Met polymorphism (rs4680; Val^158Met, corresponding to position 108 in *S-COMT*) alters enzyme thermostability and catalytic activity. Biochemical studies have shown that the Met(108/158) variant is more thermolabile, whereas the Val variant is associated with higher stability and activity, with Met carriers exhibiting approximately 3–4-fold lower COMT activity [49,50]. Functional imaging and neurocognitive studies indicate that this variation modulates the PFC dopamine tone and network efficiency, with Met carriers often showing more efficient recruitment of prefrontal resources during executive tasks and Val carriers displaying a more stress-reactive pattern of activation, a profile sometimes summarized as the “worrier/warrior” model [51,52,53].

Pharmacological challenge studies support an inverted U-shaped relationship between PFC dopamine levels and cognition. The effect of dopaminergic agents such as d-amphetamine on working memory and PFC activation has been shown to depend on rs4680, with greater improvement typically observed in Val/Val individuals and possible worsening or neutral effects in Met/Met carriers, although not all replication attempts have confirmed a robust *COMT* × amphetamine interaction at the behavioral level [54,55]. Similarly, centrally acting COMT inhibitors (e.g., tolcapone) can improve executive function and sensory gating predominantly in Val/Val individuals, while effects in Met/Met carriers are smaller or absent, consistent with movement along the inverted U-curve rather than a simple linear relationship [56,57]. Beyond rs4680, common *COMT* haplotypes that integrate synonymous variants alter mRNA structure, protein expression, and pain sensitivity, with lower-activity haplotypes associated with increased pain sensitivity [58]. Epigenetic regulation (COMT methylation in the context of stress), interactions with other dopaminergic genes (e.g., *DAT1*), and sex-specific effects related to hormonal status further modulate COMT-related phenotypes and PFC efficiency [59,60,61].

Beyond rs4680, several *COMT* haplotypes that integrate additional synonymous variants modulate enzyme expression and pain sensitivity, but cannabinoid-specific data are currently lacking [58].

In the context of cannabis, an initial cohort study suggested a *COMT Val158Met* × cannabis interaction for psychosis risk; however, a subsequent meta-analysis did not confirm a robust or consistent effect, indicating the heterogeneity and likely context dependence of any *COMT* × cannabis interaction [62]. Together with the existence of multiple functionally distinct *COMT* haplotypes, this argues against using rs4680 alone as a biomarker for MM stratification. In this review, we therefore treat COMT as a mechanistic dopaminergic modifier rather than a clinically actionable PGx marker [62].

#### 3.1.6. PGx/DDI—Practical Basics in Cannabinoid Therapy

**Figure 5:**
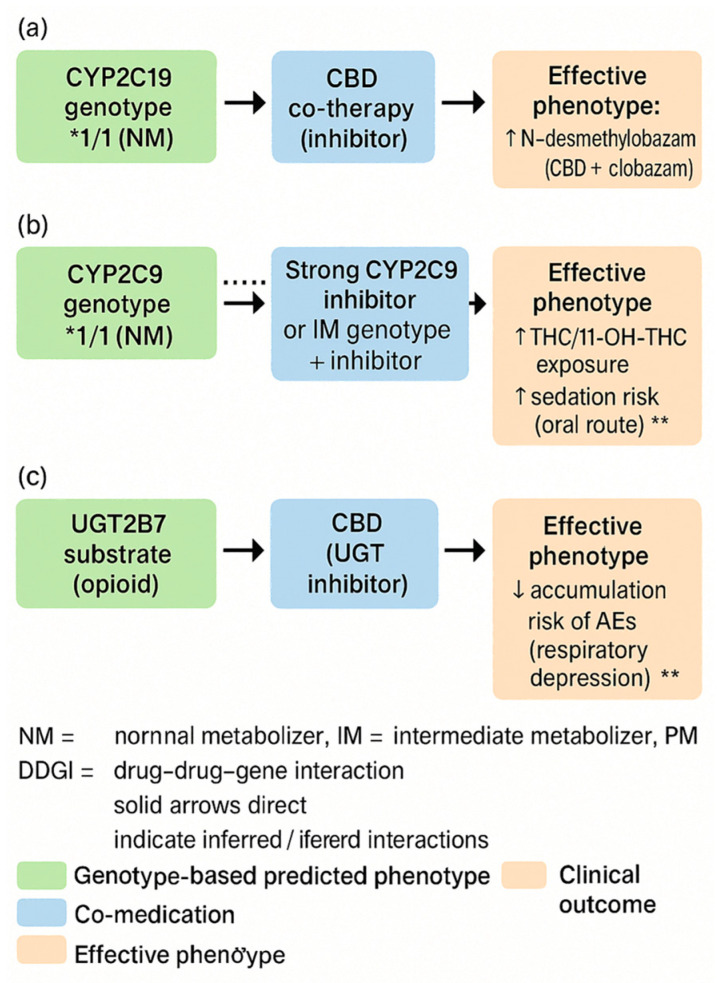
Phenoconversion and drug–drug–gene interactions (DDGIs) in cannabinoid therapy. (**a**) Schematic illustration of phenoconversion in patients treated with cannabinoids. A genetically predicted metaboliser status (e.g., normal metaboliser) can be converted into a functional poor, intermediate, or ultrarapid metaboliser phenotype by comedications (enzyme inhibitors/inducers), comorbidities, or environmental factors, leading to altered cannabinoid exposure and clinical response. (**b**) Conceptual overview of DDGIs in cannabinoid therapy. Cannabinoids, concomitant drugs, and pharmacogenetic variants in drug-metabolising enzymes (e.g., CYP450 isoforms) interact to increase or decrease cannabinoid plasma levels, which may impact efficacy, adverse effects, and dosing requirements. (**c**) Clinical consequences of phenoconversion and DDGIs, showing how altered metabolic phenotype changes cannabinoid plasma levels and leads to differences in efficacy and risk of adverse effects. * statistically significant difference at *p* < 0.05; ** statistically highly significant difference at *p* < 0.01. The dotted line represents the reference value (expected exposure with the genotype-predicted phenotype, i.e., no phenoconversion/no DDGI).

Adverse drug reactions and loss of effectiveness are among the most common, preventable causes of morbidity. Two key, intersecting determinants of response variability are drug–drug interactions (DDIs) and pharmacogenomics (PGx). The strongest decision-making basis is obtained when both dimensions are considered together, as DDIs can exacerbate, suppress, or reverse the phenotype predicted from the genotype (known as phenoconversion), leading to clinically specific DDGIs [22]. To visually summarize how co-medications can modify genotype-based phenotype predictions, Figure 5 illustrates phenoconversion and typical DDGI scenarios relevant to medical marijuana therapy. Examples include CBD–CYP2C19/3A with clobazam, CYP2C9–THC (oral route), and UGT2B7-related interactions, which together explain clinically meaningful variability in exposure and adverse effects.

Regarding the implications for medical marijuana (MM), in clinical practice the primary factors are as follows: THC–*CYP2C9* (increased exposure and sedation in *2/*3 carriers), CBD–CYP2C19/3A (inhibition with the risk of increased exposure to co-administered drugs, especially benzodiazepine derivatives), and UGT2B7 (possible modifications of glucuronidation), as well as the role of the *ABCB1* transporter in CNS availability and *AKT1/COMT* polymorphisms as modifiers of neuropsychiatric risk. PGx assessment must be contextual, taking into account current pharmacotherapy and potential phenoconversion [22].

Regarding the demonstration of evidence, the PREPARE study showed that preemptive panel testing (12 genes) integrated into the prescribing process reduces the frequency of clinically significant ADRs—evidence of the utility of systemic PGx when results are embedded in the EHR and used in practice [23]. Paradigms from other fields relevant to patients taking MM in polytherapy include the following: *CYP2C19*-dependent antiplatelet therapy (POPular Genetics and TAILOR-PCI) [63], warfarin dosing (EU-PACT, COAG, and GIFT) [64], *SLCO1B1* and statins [65], co-prescribing opioids and benzodiazepines [66], and statin exposure DDI with macrolides (CYP3A) [67]. Phenoconversion has been formalized in CPT models and confirmed prospectively (e.g., CYP2C19 inhibition), which necessitates always interpreting PGx in light of the current medication list; a classic example is the “from UM to PM” conversion of *CYP2D6* under the influence of an inhibitor [22,68].

The PGx/DDI-aware care model can be represented as follows:

(1) Panel testing and data in the EHR with clear phenotypes and ordering rules (CPIC; consistency with institutional policies) [23,69,70].
(2) A CDS with real-time phenotype correction (detection of strong inhibitors/inducers and “from IM to PM” assignment, etc.), with attention to alert ergonomics [22,71].
(3) Regarding clinical priorities and equitable access, older patients and those on polypharmacy require a full DDI-PGx review; audits of inequalities in testing and follow-up are needed [72,73].

Practical basics applied to the use of MM can be distinguished as follows.

(1) *CYP2C9*–THC: *2/*3 → ↑ exposure/11-OH-THC (orally); a lower starting dose and slower titration should be considered (description in Section 4.1).
(2) CBD–CYP2C19/3A–clobazam: inhibition → ↑ N-CLB; IM/PM require careful titration and monitoring.
(3) UGT1A9/UGT2B7: inhibition by CBD (THC to a lesser extent) → possible DDI with opioids (description in Section 3.1.2).
(4) *ABCB1* rs2235048: modulation of acute reactions after inhalation (description in Section 4.1).
(5) *AKT1* rs2494732: ↑ sensitivity to acute psychotomimetic effects; COMT without consistent interaction (meta-analysis) (description in Section 4.2).
(6) Supply route and PGx/DDI phenotype: Differences between routes/formulations (including first-pass effect and the role of UGT/CYP) can interact with PGx/DDI and should be considered during titration [22,23,63,64,65,66,69,72].

In summary, it can be stated that the best benefits come from integration: preventive PGx, ongoing DDI monitoring, and dynamic phenotype adjustment in CDS, combined with careful titration and safety monitoring in MM [23]. The practical implications of PGx/DDI are summarized in Table 2.

#### 3.1.7. *AKT1* as a Downstream Modulator of Cannabinoid Signaling

*AKT1* encodes a serine/threonine kinase which is a central effector of the PI3K–AKT–mTOR pathway and integrates multiple receptor-driven signals, including CB1/CB2-coupled cascades. Through its effects on neuronal survival, synaptic plasticity, and network excitability, altered AKT1 activity can modulate dopaminergic and glutamatergic circuit function and has been implicated in the pathophysiology of psychosis and related neuropsychiatric phenotypes.

Translational work shows that the *AKT1* rs2494732 variant modulates the acute psychotomimetic response to THC and interacts with cannabis exposure in determining psychosis risk. In a cohort of cannabis users, the rs2494732 genotype predicted the exacerbation of acute psychotic symptoms after naturally smoked cannabis, and an independent case–control study demonstrated a gene × cannabis interaction for first-episode psychosis, with higher risk in carriers of the risk genotype [10]. Together with convergent mechanistic data, this makes AKT1 one of the most promising pharmacodynamic markers for neuropsychiatric safety stratification in MM users. However, effect sizes are moderate and context-dependent, and no formal dosing or product-selection guidelines based on AKT1 status are currently available; we therefore treat AKT1 as a clinically relevant risk modifier rather than a fully validated standalone test for MM personalization at this stage [63].

## 4. Medical Marijuana in the Context of Genetics—A Review of Clinical Studies

Medical marijuana (MM)—defined as a range of standardized preparations containing Δ^9^-tetrahydrocannabinol (THC), cannabidiol (CBD), or their mixtures—exhibits significant inter-individual variability in efficacy and safety. More and more data indicates that some of this variability is due to genetic factors, including drug-metabolizing enzymes (CYP2C9, CYP2C19, and CYP3A), UGT transferases (UGT1A9/UGT2B7), transporters (ABCB1), and elements of the endocannabinoid system (CNR1/CNR2, FAAH, and AKT1). The aim of this literature review is to provide a synthetic overview of clinical studies of correlations between cannabis use and different genetic variants in humans (including pharmacokinetic, association, and intervention studies).

### 4.1. Pharmacokinetics and Pharmacodynamics—A PGx- and DDI-Oriented Approach

#### 4.1.1. CYP Enzymes (CYP2C9, CYP2C19, and CYP3A)

A typical, prospective experiment in healthy volunteers showed that the CYP2C9*3 variant causes a ~3-fold increase in THC exposure (AUC) and a decrease in the AUC of the acidic metabolite, which was clinically associated with greater sedation after oral THC. This is one of the strongest clinical links connecting genotype to cannabinoid response [7].

CBD is an inhibitor of CYP2C19 and CYP3A4, which is particularly significant in clinical practice for patients with epilepsy taking clobazam—a drug whose active metabolite, *N*-desmethylclobazam (N-CLB), is formed and further metabolized with the involvement of these isoenzymes. Preclinical and translational data support this mechanism and explain why the combination of CBD and clobazam can be more effective but also more likely to cause side effects in slow metabolizers [8].

#### 4.1.2. UGT Transferases

Modern research in human models has shown that the main cannabinoids (THC and CBD) inhibit key UGT isoforms (including UGT1A9 and UGT2B7), which potentially modifies the clearance of other glucuronidated drugs and may contribute to the clinical response to MM. Although these are primarily mechanistic data, their clinical significance is beginning to be described in patient studies [8].

#### 4.1.3. Transporters (*ABCB1*)

*ABCB1* polymorphisms modulate drug absorption; a controlled study involving young adults demonstrated that the rs2235048 variant influences acute psychophysiological responses to inhaled cannabis, suggesting a possible role for P-gp in THC/CBD availability in the CNS [9]. We focus on rs2235048 because it has been directly studied in the context of acute responses to inhaled cannabis, other common ABCB1 variants (e.g., rs1045642, rs2032582, and rs1128503) are known to influence P-gp function and CNS drug disposition. To date, however, no consistent associations between these polymorphisms and the clinical response to MM have been demonstrated, and further studies are needed before they can be considered for routine stratification.

#### 4.1.4. Receptors and the Endocannabinoid System (*CNR1*/*CNR2*, *FAAH*, and *AKT1*)

Clinical data indicate a significant role for *AKT1* in sensitivity to the acute psychotomimetic effects of THC and in the risk of psychosis in users. In a translational study, the *AKT1* genotype rs2494732 predicted the exacerbation of acute psychotic symptoms after naturally smoked cannabis, and an independent case–control study showed an interaction between *AKT1* and cannabis use in the risk of first-episode psychosis [10].

Regarding *COMT* Val158Met, a meta-analysis of observational studies did not confirm a consistent interaction with cannabis use in generating psychotic symptoms (an effect only observed in case-only designs with lower reliability) [11].

Several common *CNR1* single-nucleotide polymorphisms, most notably the synonymous rs1049353 (1359G/A, p.Thr453Thr) and variants located in promoter and intronic regions, have been investigated in candidate-gene studies of neuropsychiatric, metabolic, and pain-related phenotypes, as well as MM use and dependence; however, the reported associations are heterogeneous, and many have failed to replicate, suggesting small or context-dependent effects [39]. Likewise, the functional *CNR2* variant rs35761398 (Q63R) has been linked to immune response modulation and susceptibility to inflammatory and neuroinflammatory conditions, but here too the direction and magnitude of effects vary across cohorts and populations [39]. To date, no robust, clinically actionable relationship between *CNR1/CNR2* genotype and medical MM response has been established, and these markers are not included in current MM pharmacogenomic decision frameworks [39].

### 4.2. Results of the Clinical Trial Review

Based on a literature review of clinical studies, the following reports were identified:

(1) Neuropsychiatric Effects and Safety

Studies published in *Translational Psychiatry* and *Biological Psychiatry* provide data suggesting that the *AKT1* variant rs2494732 (particularly the C/C genotype) increases sensitivity to the acute psychotic effects of THC and modifies the risk of psychosis with frequent use. This has obvious implications for qualifying patients for THC-rich product therapy (e.g., avoiding high doses in C/C carriers) [10].

In light of a meta-analysis published in *PLOS ONE*, the interaction between *COMT* Val158Met and cannabis is not sufficiently documented, which tempers previous hypotheses and suggests that *COMT* testing should not be routinely used for risk stratification in MM [11].

(2) Chronic pain—pharmacogenetics of response to MM

In a large observational study of 600 patients treated with standardized cannabis preparations, a candidate gene panel analysis revealed that polymorphisms in *ABCB1*, *TRPV1*, and *UGT2B7* were associated with a greater decrease in pain intensity. The result suggests an additive polygenic effect—patients with a “favorable” allele system experienced greater clinical benefit. This is the most extensive clinical study to date linking genetics with the analgesic efficacy of MM [74].

Additionally, a controlled experiment involving young adults demonstrated the influence of the *ABCB1* rs2235048 polymorphism on acute responses to THC, which may also be relevant to the adverse effect profile in patients requiring a rapid analgesic effect [9].

(3) Drug-resistant epilepsy—the intersection of pharmacogenetics and drug interactions

Numerous data indicate that the effectiveness of CBD in Dravet/Lennox–Gastaut syndrome increases in the presence of clobazam, but CYP2C19 (the main N-CLB pathway) and CBD’s inhibitory effect on CYP2C19/3A determine a high risk of N-CLB accumulation and adverse effects—especially in slow metabolizers. The applications were supported by preclinical studies and pharmacokinetic modeling, as well as clinical observations [8].

In patients receiving clobazam—regardless of CBD—the *CYP2C19* genotype strongly determines N-CLB concentrations (PM >> IM/EM), which is directly useful for dose titration and safety monitoring in CBD therapy [75].

(4) Pharmacokinetics of THC/CBD—Implications for Genotype-Dependent Dosing

A study published in *Clinical Pharmacology & Therapeutics* found that the CYP2C9*3 variant significantly increases THC exposure after oral administration; clinically, this suggests the need for more cautious titration and lower initial doses in *3 homozygotes/heterozygotes [7].

A cross-over study published in the *Journal of Analytical Toxicology* showed significant differences in Cmax/CBD and THC detection depending on the route/formulation, which may interact with genetic factors (e.g., the different roles of first-pass metabolism and UGT/CYP) [76].

#### 4.2.1. Clinical Implications (Proposed Practical Algorithm)

(1) It is proposed to consider limiting or avoiding high doses of THC in patients with positive family histories of psychosis; in situations of increased risk, targeted testing for *AKT1* rs2494732 may be useful (clinical evidence of G×E) [10].
(2) In light of data from 600 patients, genetic factors (*ABCB1/TRPV1/UGT2B7*) may differentiate the response; in practice, consider documenting the phenotypic response and (when available) panel pharmacogenetic testing during long-term MM treatment [74].
(3) Epilepsy—safety and efficacy:
  • When combining CBD with clobazam, it is recommended to start with lower doses of clobazam (or a slower titration) in individuals who are *CYP2C19* PM/IM and to routinely monitor N-CLB levels and adverse effects [75].
  • It should be noted that CBD inhibits CYP2C19/3A—the effect can be intensified by the PM genotype and other inhibitors [8].
(4) In patients with CYP2C9*3, lower starting doses and slower escalation should be considered; educate about increased sedation [7,77].

#### 4.2.2. Limitations of Current Data

Although there is strong evidence for *AKT1* (psychotomimetic effects) and *CYP2C9/CYP2C19* (PK/safety), many observations (e.g., *ABCB1/TRPV1/UGT2B7* in pain) come from non-randomized observational studies suggesting correlation alone and further independent replication is necessary. For *COMT*, there is no consistent clinical interaction in the meta-analysis. Pragmatic RCTs with embedded pharmacogenetic frameworks and clinical trials are needed [74].

In summary, it can be stated that the pharmacogenetics of MM is entering a stage of clinical maturity in three areas: (1) safety and exposure—*CYP2C9* (THC) and *CYP2C19* (CBD–clobazam interaction); (2) neuropsychiatric risk—*AKT1* as a moderately strong modifier of individual susceptibility; (3) analgesic efficacy—preliminary but promising multigene signals (*ABCB1/TRPV1/UGT2B7*). Integrating PGx testing with careful titration and monitoring for adverse events can realistically improve the benefit–risk profile of MM therapy today [10].

**Figure 6:**
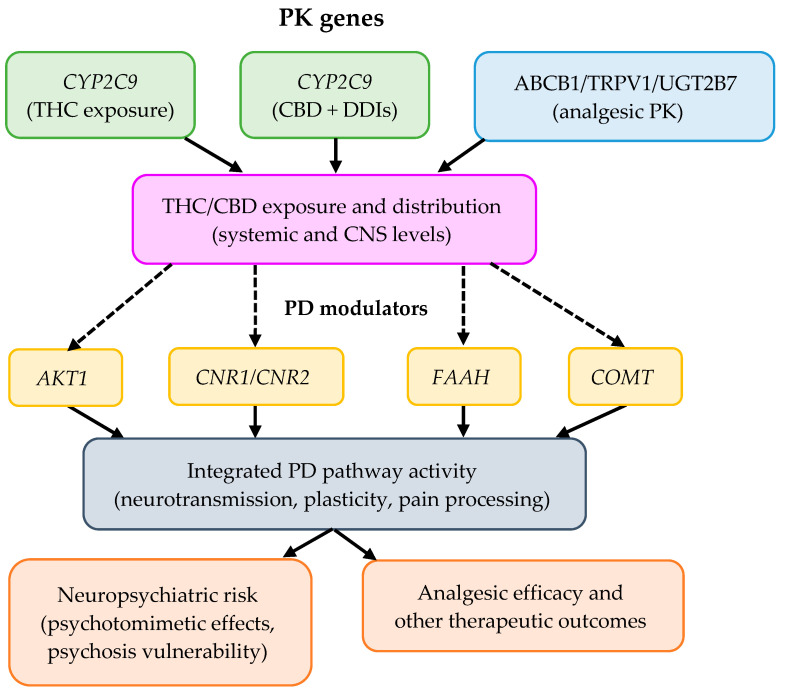
Conceptual PK-PD model of the proposed working hypothesis on gene–gene interactions influencing cannabinoid response.

To better conceptualize the proposed framework of gene–gene interactions, Figure 6 presents a working hypothesis model integrating pharmacokinetic and signaling components relevant to cannabinoid response. Figure 6 summarizes the hypothetical interaction network between PK genes (*CYP2C9* and *CYP2C19*) and PD modulators (*AKT1, CNR1/CNR2, FAAH, COMT,* and *ABCB1/TRPV1/UGT2B7*) and how they converge in exposure, neuropsychiatric risk, and analgesic efficacy. Dotted lines represent indirect or inferred interactions requiring empirical validation.

## 5. Epigenetic Pathways of Cannabinoid Action

Cannabinoids, both endogenous (AEA, 2-AG) and exogenous (including Δ^9^-tetrahydrocannabinol, THC, and cannabidiol, CBD), influence gene expression through a network of connections between CB1/CB2 receptor signaling and epigenetic mechanisms: DNA methylation, histone modifications, and regulation by non-coding RNA. The consequences of these interactions are cell-type and developmental context-dependent; they include plastic, and sometimes long-lasting, transcriptional changes associated with neurodevelopment, immunity, neuropathic pain, fertility, and oncogenesis [78].

### 5.1. The Endocannabinoid System and the Framework of Epigenetics

The endocannabinoid system (ECS) is composed of CB1/CB2 receptors, their endogenous ligands, and biosynthesis and degradation enzymes; receptor signaling integrates with epigenetic regulation, influencing transcriptional programs in nervous and immune tissues. Reviews present a strict, bidirectional relationship between the ECS and epigenome modifications in health and disease [79].

**Figure 7:**
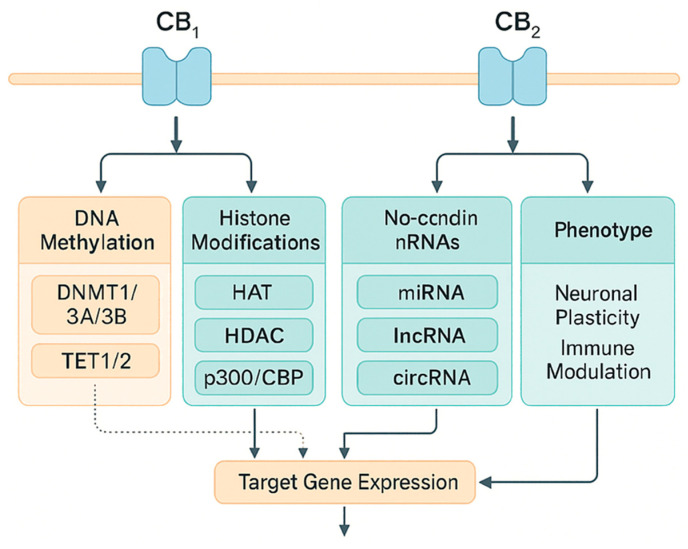
Integration of CB1/CB2 signaling with epigenetic mechanisms: DNA methylation, histone modifications, and non-coding RNAs. Integration of CB1/CB2 signaling with epigenetic mechanisms: DNA methylation, histone modifications, and non-coding RNAs. Legend: DNMT: DNA methyltransferase; TET: ten-eleven translocation methylcytosine dioxygenase; HAT: histone acetyltransferase; HDAC: histone deacetylase; miRNA: microRNA; IncRNA: long non-coding RNA. Solid arrows indicate experimentally supported regulatory effects (up- or downregulation of the target process, as indicated by arrowheads). Dotted arrows indicate inferred or indirect links requiring further validation. CBP: CREB-binding protein, a transcriptional co-activator with histone acetyltransferase activity. circRNA: circular RNA, a class of non-coding RNAs that regulate gene expression (e.g., by interacting with miRNAs and epigenetic pathways).

Epigenetics includes DNA methylation (DNMT/TET activity), histone modifications (HAT/HDAC action and acyl readers), and regulation by miRNA/lncRNA; these processes determine chromatin accessibility and the stability of transcriptional states. Internal histone acylations, p300/CBP, inherit and propagate acetylation signals within the nucleosome, which is fundamental for epigenetic memory [80]. To integrate receptor signaling with chromatin-level regulation, Figure 7 presents a layered model linking CB1/CB2 pathways to three epigenetic axes—DNA methylation (DNMT/TET), histone modifications (HAT/HDAC/p300–CBP), and non-coding RNAs (miRNA/lncRNA). The scheme summarizes how CB1/CB2-driven cascades (e.g., ERK/CREB) converge on these regulators to shape target-gene expression and downstream phenotypes; dotted arrows mark inferred links that require further validation.

### 5.2. Epigenetic Mechanisms Activated by Cannabinoids

#### 5.2.1. DNA Methylation

Activation or blockade of the ECS can modulate the expression of methylating/demethylating enzymes and the methylation patterns of target gene promoters. In controlled human exposure studies, concentration-dependent changes in the expression of DNMT and TET were observed in peripheral blood mononuclear cells, suggesting a direct impact of THC on the methylation machinery [13].

At the level of neural tissues, brain development correlates with specific changes in the expression and methylation of the *CNR1* (*CB1*) gene, indicating that epigenetics co-determines the transcriptional maturation of the receptor in cortico-hippocampal regions [81].

#### 5.2.2. Histone Modifications and Chromatin Architecture

After peripheral nerve damage, “bivalent” histone marks (simultaneous H3K4me3/H3K27me3) are observed in sensory neurons at the *Cnr2* gene; these marks precede the increase in CB2 receptor expression and are co-responsible for the analgesic effect of CB2 activation. This mechanism links the epigenome to sensory function and pain transmission [82].

At a general level, the acetylating enzymes p300/CBP can read and replicate histone tail acetylation within the same nucleosome, locally destabilizing H2A–H2B and promoting transcription, which is a likely site for the integration of *CB1/CB2* signals (e.g., via ERK/CREB) with epigenetic memory [80,83].

#### 5.2.3. Non-Coding RNA (miRNA, lncRNA)

THC and CBD modulate miRNA and longer ncRNA profiles, linking cannabinoid signaling to translational regulation of expression. In monocytes/myeloid suppressor cells, THC reprograms the miRNA set and their target pathways, and in inflammation models, CBD alters histone methylation patterns and miRNA/lncRNA expression, suppressing pro-inflammatory transcription [84].

### 5.3. Exogenous Cannabinoids: From Receptor Signaling to the Epigenome

#### 5.3.1. Δ^9^-Tetrahydrocannabinol (THC)

In human PBMC samples, exposure to THC (within a concentration range reflecting use) modifies the expression of DNMT/TET and inflammatory markers, indicating a potential dose-dependent impact on DNA methylation and the immune microenvironment [13].

In population-based association studies, cannabis use was linked to differences in DNA methylation in blood and accompanying changes in cognitive function, which supports the concept of an epigenetic footprint of use style on systemic transcriptional parameters [85].

In oocytes exposed to THC in vitro, the dominant effect was DNA methylation changes, with limited impact on the miRNA profile, suggesting selective sensitivity of the female gamete epigenome [86].

#### 5.3.2. Cannabidiol (CBD)

CBD exhibits complex immunomodulatory effects, partially through changes in histone methylation and non-coding RNA expression in lymphocytes, while simultaneously “reversing” pro-inflammatory transcriptional signatures. In studies on autoimmune models, suppression of proinflammatory axes involving miRNAs and lncRNAs was observed [87].

From a developmental perspective, exposure of juvenile animals to CBD modified DNA methylation patterns and was associated with an anxious phenotype, highlighting the sensitivity of the epigenome during critical periods [88].

### 5.4. Developmental Windows and Intergenerational Transmission

#### 5.4.1. Prenatal Exposure

Fetal–placental exposure to THC is associated with changes in the DNA methylation of neurodevelopmental genes in the placenta and fetal tissues in humans and primates, potentially programming the offspring’s long-term behavioral trajectories. Data from cohort and translational studies (macaques) consistently point to epigenetic modifications as an intermediary mechanism [15].

#### 5.4.2. Male Line: Sperm Epigenome

A study published in *Epigenetics* showed that exposure to cannabinoids (in humans—cannabis use; in rats—THC) is associated with extensive DNA methylation changes in sperm (thousands of CpG sites), including developmental genes [89].

Subsequent studies confirmed that some of these changes can be reduced after a period of abstinence (≈one spermatogenesis cycle), suggesting a partial reversibility of the epigenetic signature in male gametes [14].

In rodents, the maintenance of selected methylation changes in offspring and their association with organ phenotypes (e.g., cardiomegaly) have also been demonstrated, which supports the hypothesis of epigenetic transmission of paternal exposure effects [90].

**Figure 8:**
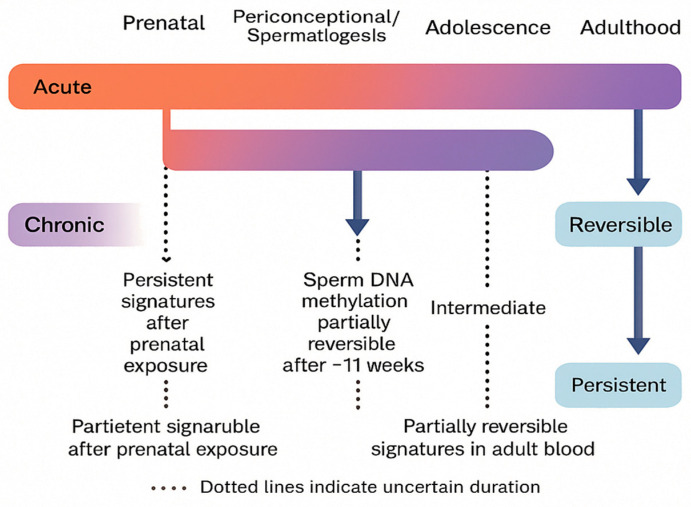
Reversibility and persistence of cannabinoid-related epigenetic changes across developmental windows.

To contextualize exposure timing with epigenetic outcomes, Figure 8 maps acute and chronic cannabinoid exposures onto key developmental windows (prenatal, periconceptional/spermatogenesis, adolescence, and adulthood). The timeline highlights partially reversible signatures in adult blood, the ~11-week reversibility of male germline marks across a spermatogenesis cycle, and the greater persistence of prenatal exposure signatures; dotted lines denote uncertainty in duration.

### 5.5. Diseases and Clinical Contexts

The aforementioned “bivalent” histone marks in sensory neurons after nerve damage pave the way for CB2 overexpression, and CB2 activation suppresses nociceptive conduction which justifies epigenetic targets for pain therapy (e.g., modulation of the H3K4me3/H3K27me3 landscape) [82].

Cannabinoids influence the metabolic and expression phenotype of cancer cells, including through epigenetic pathways; the conceptual framework links GPCR signaling (CB1/CB2) with chromatin remodeling and transcriptional reprogramming, which are hallmarks of cancer. Although translational findings require caution, the p300/CBP pathways and acylation readers appear to be potential therapeutic targets [83,91].

Analyses of the blood epigenome indicate that cannabis-use patterns are associated with differences in DNA methylation and differences in verbal learning; these observations are consistent with the hypothesis that environmental factors (use) leave a detectable epigenetic mark [85].

### 5.6. Methodological and Therapeutic Implications

#### 5.6.1. Tissue and Temporal Specificity

The conclusions depend on the cell type and developmental window (gametes, placenta, PBMC, and neurons), and the heterogeneity of the preparations (e.g., blood) can dilute the signal; therefore, combining WGBS/RRBS with expression and miRNA profiling is recommended. Cohort studies should control for confounding factors (tobacco, alcohol, and diet) [89].

#### 5.6.2. Pharmacological Effects

Epigenetic nodes (DNMT/TET; p300/CBP; HDAC) integrate CB1/CB2 signals. In oncology and neuropsychopharmacology, small-molecule p300/CBP inhibitors/degraders and acyl reader modulators are being developed, which provides a framework for combining epigenetic therapies with ECS-modulating drugs—although this requires rigorous safety and efficacy studies [83,92].

In summary, the results of clinical, population, and experimental studies consistently indicate that cannabinoid signaling intersects with epigenetic regulation at multiple levels: from the DNA methylome, to the histone modification landscape, to non-coding RNA networks. These effects can be partially reversible (e.g., in sperm after abstinence), but during critical windows (pregnancy and puberty), they are associated with long-term phenotypic programming. The priorities remain mapping causality (from the CB1/CB2 signal to a specific epigenetic enzyme), validating translatability between tissues and species, and assessing the risk/benefit ratio in therapeutic applications [14].

## 6. Reversibility and Persistence of Epigenetic Changes

The human epigenome remains a dynamic melting pot of genetic and environmental influences. Changes such as DNA methylation, histone modifications, or regulation by non-coding RNA can be both reversible and persistent over longer time periods and even under certain conditions across generations. Identifying which epimutations are plastic and which are fixed is crucial for assessing the safety and efficacy of therapeutic interventions, including MM-based therapies. In recent years, reviews and conceptualizations have highlighted that individual epigenetic variability is a significant component of clinical response differentiation and a potential target for treatment personalization. At the same time, the translation of epigenetic biomarkers into clinical practice is developing, particularly in oncology, but also in chronic diseases and in assessing the impact of environmental exposures [83,93].

### 6.1. Molecular Mechanisms: How Cannabinoids Interact with the Epigenome

Phytocannabinoids (e.g., Δ^9^-tetrahydrocannabinol, THC; cannabidiol, CBD) modulate the endocannabinoid system through *CB1/CB2* receptors and a range of off-target signaling pathways. These signals can influence the function of epigenetic “writers,” “readers,” and “erasers” (DNMT, TET, HDAC/HAT, and 5mC-binding proteins), affecting chromatin accessibility and gene expression. Transcriptomic and epigenetic studies in human brain tissues indicate that the expression of the *CNR1* gene (CB1) correlates with age and methylation patterns at specific loci (e.g., cg02498983), and exposure to THC may be associated with deregulation of *CNR1* expression—providing indirect evidence that cannabinoid signaling and epigenetic regulation are linked. Additionally, experimental data for CBD suggest that it can modulate the epigenome and behavioral phenotypes during brain development, highlighting the potential impact of cannabinoids on epigenetic enzymes and synaptic plasticity [81].

### 6.2. Evidence in Humans: Blood Methylation, Epigenetic Aging, and Exposure Signatures

In epigenome-wide association studies (EWASs) using blood samples from adults, reproducible associations have been found between cannabis use and differences in DNA methylation. In EWAS analyses of blood, most cannabis-associated CpG sites showed small absolute methylation differences (typically Δβ < 5%) between users and non-users, consistent with other environmental exposures. Nonetheless, several signals were replicated across time points and cohorts, supporting their robustness as exposure markers rather than large-effect determinants of disease. A summary of selected cannabis-associated DNA methylation signatures, including effect sizes and reversibility, is presented in Table 3 [14,15,89,90]. A multi-time-point EWAS (CARDIA; two time points) identified 201 methylation markers associated with recent and cumulative marijuana use and 198 differentially methylated regions; some of the effects were replicated over time and in independent analyses, indicating consistency in the exposure signatures. Concurrently, a large trans-ancestral meta-analysis (N ≈ 9,436) for “ever vs. never” revealed specific CpG sites associated with cannabis use, laying the groundwork for the development of methylation biomarkers of exposure. In a separate study of young adults, marijuana use was associated with an accelerated epigenetic age estimated by the GrimAge measure, with effects dependent on the intensity and recency of exposure. Together, these data suggest that some of the changes are short-lived (related to “recent” exposure), while others reflect a cumulative effect [12].

An interesting aspect is the persistence of methylation signatures in former users. A study of the epigenome in older adults (the CanCOLD analysis) found that many methylation signals associated with cannabis smoking persist despite cessation of use, with heterogeneity of effects at the gene and pathway levels. This strengthens the thesis that a “permanent” component coexists with a reversible component [12]. Further research in this area could lead to better understanding of current mechanisms as well as breakthroughs for new long-term therapeutic effects.

### 6.3. Reversibility and Persistence: Conclusions from Gametes and Somatic Lines

Cannabis/THC exposure in men was associated with both hyper- and hypomethylation across thousands of CpGs in sperm, with partial normalization (reduced effect sizes) after ~11 weeks of abstinence; however, a subset of loci remained differentially methylated, indicating a mixture of reversible and more persistent marks. A similar phenomenon (decrease in the amplitude of differences after a “wash-out” period) was observed in a rat model (exposure to cannabis extract). However, some CpG sites appear to maintain differences despite abstinence, suggesting that the selected epialterations are more persistent or decay more slowly [90].

On the other hand, peripheral blood population study results indicate that some methylation signatures are “here and now” exposure markers (e.g., strongly associated with recent use), while others are long-lasting and can persist after exposure has ceased. This dichotomy corresponds to modern models of epigenome stability: marks can be dynamically “written” and “erased,” but their persistence depends on factors such as cellular context, age, exposure dose/window, and the involvement of the transcription–chromatin feedback loop [94].

### 6.4. Transgenerationality: Knowns and Unknowns

Rodent models show that exposing fathers to THC or cannabis extract can be associated with DNA methylation modifications in sperm, which are partially passed on to offspring tissues along with changes in expression and developmental phenotypes (e.g., cardiomegaly). At the same time, methodological reviews highlight strong limitations in extrapolating to humans due to global epigenetic reprogramming after fertilization and barriers to stable epigenetic transmission. In summary, intergenerational epigenetic effects after cannabis exposure are biologically plausible in models, but in humans, they require much stronger evidence with rigorous consideration of genetics and the environment [90].

### 6.5. Clinical Implications for Medical Marijuana

#### 6.5.1. Epigenetic Safety

Data from gametes suggest that abstinence for approximately one spermatogenesis cycle can limit some methylation changes in men planning to have children, which has practical implications for pre-conception counseling for patients using MM (e.g., for chronic pain). At the same time, blood signatures can persist longer in some individuals, indicating the need for long-term monitoring of exposure and potential side effects [12,14,95].

#### 6.5.2. Epigenetic Age and Comorbidities

The relationship between cannabis use and accelerated epigenetic age (GrimAge) suggests a possible impact on cardiovascular risk and overall lifespan, although causality and modifiers (e.g., alcohol, tobacco) remain debated. In clinical practice, this information can support individualized risk or benefit assessment, for example, in patients with multimorbidity [96].

#### 6.5.3. Pharmacogenomics and Response Prediction

Although validated, routine “epigenetic selection tests” for dose or chemotype of MM are still lacking, several premises support their development: (i) reproducible methylation signatures associated with cannabis use; (ii) known epigenetically regulated pain/immune pathway gene networks; (iii) mature frameworks for evaluating epigenetic biomarkers (multi-phase design, validation, and clinical utility). Applications may include exposure classification (compliance), stratification of adverse event risk (e.g., mood disorders), and in the future, prediction of the analgesic response to specific preparations (THC/CBD) through integrated DNAm signatures in leukocytes. However, prospective validation, calibration against co-exposure (nicotine/alcohol/medications), and avoiding excessive inference with weak single CpG effects are prerequisites [97].

### 6.6. Proposed Epigenomics-Based Framework for Treatment Personalization

The following stages of epigenome-based treatment personalization are proposed:

(1) Preliminary clinical qualification—indications with confirmed efficacy (e.g., neuropathic pain, spasticity, and selected epileptic syndromes), with an assessment of risk factors.
(2) Exposure and risk profile—an “exposure” DNAm panel derived from replicable EWAS (e.g., signatures associated with cannabis use and epigenetic aging), for informational purposes only and not a substitute for clinical monitoring.
(3) Stratification and drug selection—decision on chemotype and route of administration, taking into account the patient profile (age, comorbidities, and interaction potential), with a plan for dose reduction when planning conception (in men).
(4) Monitoring response and safety—linking clinical indicators (pain scales and function) with longitudinal blood draws for exploratory DNAm profiling (research/”learning health system”) to build predictors.
(5) Validation and implementation—applying a five-phase biomarker assessment framework (first: pre-clinical analysis; second: clinical evaluation; third: utility), in accordance with current guidelines for epigenetic biomarkers [98].

### 6.7. Limitations of Current Data

Most human studies are based on peripheral blood (proxy), with limited mapping to target tissues (brain or immune system). The effects are largely small, polygenic, or “polyepigenetic,” and the heterogeneity of methods (platforms, normalization, and corrections) makes comparisons difficult. It is also critical to separate the influence of cannabis from tobacco and other exposures, and to rigorously control for socio-environmental variables. Finally, transgenerationality in humans remains a hypothesis requiring evidence resistant to embryonic reprogramming [99].

In summary, this chapter concludes that in the context of MM, epigenetics offers two complementary avenues: (a) monitoring exposure and safety, with signatures in blood and gametes that are partially reversible after abstinence but may also have a permanent component; (b) the perspective of personalization, where epigenetic biomarkers, after appropriate validation, could assist in selecting a preparation/monitoring strategy for specific patients. Both lines of research require prospective clinical trials, multi-center replication, and multi-omic integration before they can be implemented in routine clinical practice.

## 7. Perspectives on Therapy Personalization

Personalized therapy (precision medicine) involves tailoring interventions to the diverse characteristics of the patient—biological (e.g., pharmacogenetics), phenotypic (age, sex, and comorbidities), behavioral, and environmental—in order to maximize effectiveness and minimize the risk of adverse effects. In the field of MM application, personalization takes on particular significance due to the following: (1) the complexity of the endocannabinoid system (ECS), (2) the variety of forms and routes of administration (inhalation, oromucosal, oral), (3) significant pharmacokinetic interactions, and (4) heterogeneous clinical response. In recent years, both new data from randomized trials (including in chronic pain and drug-resistant epilepsy) and real-world evidence analyses have emerged, enabling more informed design of personalization pathways [100].

### 7.1. The Biological Basis and Clinical Pharmacology of Cannabis

The endocannabinoid system includes ligands (anandamide, 2-AG), receptors (CB1/CB2), and metabolic enzymes (including FAAH and MAGL). At the drug level, phytocannabinoids play a key role—primarily Δ^9^-tetrahydrocannabinol (THC) and cannabidiol (CBD)—whose pharmacokinetics and pharmacodynamics differ depending on the route of administration and the composition of the preparation. A review in the *British Journal of Clinical Pharmacology* highlights that the bioavailability and onset of action are significantly different for inhaled versus oral/oromucosal administration, and the conversion of THC to 11-OH-THC (an active metabolite) is clinically relevant (e.g., for psychotropic and analgesic effects) [16].

#### 7.1.1. Metabolism and Individual Differences

New research in biochemistry and pharmacology indicates that the clearance of THC is dominated by cytochrome P450 isoenzymes CYP2C9 and CYP2C19 (with the involvement of CYP3A), and variability in these pathways, including *CYP2C9* polymorphisms, can modulate exposure to THC and its metabolites. This concept is supported by research in *Biochemical Pharmacology* (2024) and interaction reviews published in the *AAPS Journal*. PBPK modeling including THC/CBD confirms the complexity of CYP and UGT involvement and the variability in the contributions of individual enzymes between studies [2].

#### 7.1.2. Sex or Population Section Differences

**Figure 9:**
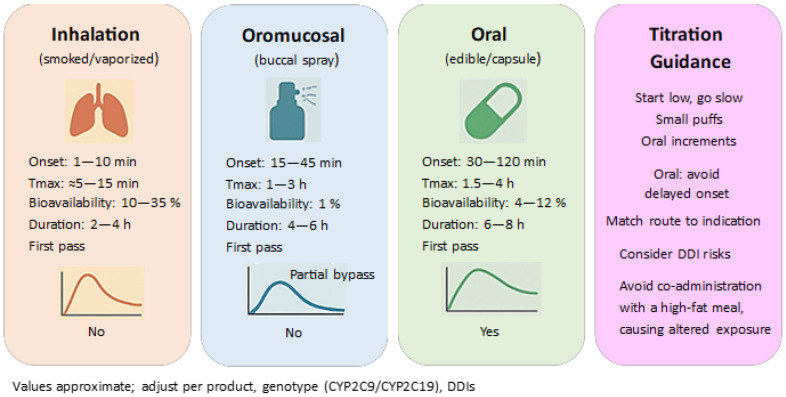
Routes of administration and pharmacokinetic profiles of THC/CBD.

Data is emerging that suggests sex differences in response to THC (differences in pharmacokinetics and subjective effects after oral THC), which has implications for dose selection and adverse event monitoring. Clinical and experimental research results (e.g., *Psychopharmacology* 2024) highlight the possibility of stronger subjective effects in women at similar concentrations, although the human literature remains inconclusive; meanwhile, toxicokinetic studies (*Journal of Analytical Toxicology*) describe differences in metabolite excretion profiles [101]. To contextualize route-dependent exposure and onset, Figure 9 summarizes pharmacokinetic profiles for inhalation, oromucosal, and oral administration (onset, Tmax, bioavailability, duration, and first-pass metabolism). This overview supports titration choices by linking route-specific PK features to practical dosing considerations in the clinical use of THC/CBD.

#### 7.1.3. The Supply Chain and Personalization

In the context of personalization, it is important to differentiate between routes of administration (e.g., oromucosal THC/CBD spray vs. capsules/oils). Direct comparisons of THC/CBD bioavailability between oromucosal and oral formulations indicate significant differences in pharmacokinetic parameters, which may necessitate different titration schemes [102].

### 7.2. Clinical Evidence in Major Indications and Implications for Personalization

#### 7.2.1. Chronic Pain

Reviews and guidelines from *BMJ Rapid Recommendations* indicate a “weak (conditional) recommendation” for a trial of non-inhalable therapy in adults with chronic pain when standard care does not provide relief. At the same time, attention is drawn to the small or moderate analgesic effects and the need to balance the benefits against the risks [16].

As of 2025, large, modern RCTs are also emerging. A study published in *Nature Medicine* involving patients with chronic lower back pain showed that a low-dose, liquid cannabis extract resulted in a clinically significant reduction in pain intensity compared to placebo and improved function—with an acceptable safety profile. While these results are promising, they apply to a specific formulation and population, so they cannot be directly extrapolated to all MM products [18].

Complementarily, a systematic review in the *Annals of Internal Medicine* (EPC/AHRQ) highlights that the analgesic effect in previous RCTs is most often small, and the quality of evidence is moderate to low; therefore, therapeutic decisions should consider patient preferences and early assessment of response and tolerance [17].

#### 7.2.2. Spasticity in Multiple Sclerosis (MS)

A meta-analysis in *JAMA Network Open* showed moderate evidence for a reduction in spasticity and associated pain in MS with the use of cannabinoids (including nabiximols—an oromucosal THC/CBD spray), with generally acceptable safety. RCT data support the use in patients with symptomatic spasticity resistant to standard pharmacotherapy, while remaining vigilant for dizziness, drowsiness, and fatigue [19].

#### 7.2.3. Drug-Resistant Epilepsy (Developmental Epilepsies)

High-quality RCTs from the *New England Journal of Medicine* and *The Lancet* confirmed the efficacy of purified CBD as an add-on treatment for Dravet syndrome and Lennox–Gastaut syndrome, leading to product registration (Epidiolex/Epidyolex) and establishing a benchmark for evidence quality in the MM area. Personalization in this indication includes, among other things, interaction control (especially with clobazam and valproic acid) and titration with monitoring of liver enzymes [20].

### 7.3. Pharmacogenetics, Drug Interactions, and Safety as Pillars of Personalization

#### 7.3.1. Pharmacogenetics (CYP)

Experimental and translational results indicate that THC is primarily metabolized by CYP2C9 and CYP2C19, and that individual differences (e.g., CYP2C9 *2/*3 variants) can affect exposure and the risk of adverse effects, which justifies more cautious titration in potential “poor metabolizers” [2].

#### 7.3.2. Drug–Drug Interactions (DDIs)

CBD inhibits CYP enzymes (especially CYP2C19 and CYP3A4), which can increase exposure to the active metabolite of clobazam (*N*-desmethylclobazam)—with the risk of excessive sedation; this phenomenon has been demonstrated preclinically and observed in clinical populations. Additionally, the combination of CBD with valproate has been associated with increased aminotransferase activity, which justifies monitoring liver function tests [8].

#### 7.3.3. Security Profile

A large data synthesis in *BMJ Open* (chronic pain) indicates that adverse events (dizziness, drowsiness, dry mouth, and nausea) are common, but severe ones are rare. Personalization requires consideration of central nervous system vulnerability (e.g., in older adults), a history of psychotic disorders (caution with high doses of THC), and cardiovascular conditions [103].

In addition to traditional clinical risk factors, the implementation of MM personalization is increasingly shaped by genomic and epigenomic signals. Based on the evidence reviewed in Section 3, Section 4, Section 5 and Section 6, we distinguish between markers that are ready for direct clinical use and those that should remain confined to research settings. The resulting implementation maturity of key pharmacogenetic and epigenetic signals in MM personalization is summarized in Table 4.

### 7.4. The “Entourage Effect”, the Chemical Composition of Cannabis, and Implications for Personalization

The hypothesis of the so-called “entourage effect” (interactions of phytocannabinoids with terpenes and flavonoids) has a solid pharmacological basis and numerous preclinical arguments, but limited and ambiguous confirmation in clinical studies; therefore, in terms of personalization, it is advisable to rely on clearly defined preparations and standardization of composition. A literature review in the *British Journal of Pharmacology* and more recent works (e.g., *Pharmaceuticals*) summarize the potential and research gaps [21].

### 7.5. Real-World Data (RWE) and Long-Term Effectiveness

Observational studies (e.g., *Pain Medicine*) and a meta-analysis of long-term studies in the *European Journal of Pain* confirm that many patients report improvement in symptom severity and quality of life, as well as a reduction in analgesic use; however, it is important to be aware of the methodological limitations (lack of randomization and risk of selection and confounding bias), which necessitates a cautious application of these findings to personalized practice [104].

### 7.6. Cannabinoids and “Opioid-Sparing”: What Does It Mean for Personalization?

An updated review in *Neuropsychopharmacology* indicates consistent preclinical evidence of an “opioid-sparing” effect, while clinical trial results are inconclusive; a *BMJ Open* analysis highlights very low certainty of evidence in the context of opioid dose reduction. In personalization, this means that the declared goal of being “opioid-sparing” requires an individual reduction plan, monitoring, and realistic communication about the expected effects [105].

### 7.7. Personalization Methods: Titration, Decision Algorithms, and Research Design

#### 7.7.1. Principles of Practical Titration

Based on BMJ guidelines and PK reviews, it is recommended to start with a low dose, prefer non-inhalable formulations (especially in the elderly and those with lung diseases), increase the dose slowly, regularly assess target outcomes (e.g., ≥30% pain reduction, improved function, sleep), and consider lack of response after 6–8 weeks as a criterion for discontinuation or change. When choosing the composition, it is worth considering a lower THC/CBD ratio (greater central safety), and in patients at risk of interaction, schemes with a lower dose of CBD and monitoring of narrow therapeutic index drug concentrations should be preferred [16].

#### 7.7.2. N-of-1 and Adaptive Statistical Approaches

For heterogeneous populations (e.g., chronic pain), N-of-1 and adaptive designs are valuable as they allow for faster identification of “responders” and optimal ranges of doses/THC/CBD ratios. The contemporary methodological literature (e.g., the *Journal of the Royal Statistical Society: Series C* on Bayesian dose optimization, as well as reviews of adaptive designs in *Frontiers in Pharmacology*) provides a framework for such approaches in personalization research, which can be translated to the clinical level (e.g., adaptive rule-based titration protocols) [106]. When constructing practical decision algorithms (Section 7.7), only markers classified as “ready for clinical implementation” in Table 4 were considered.

### 7.8. A Proposed Decision-Making Framework for Personalizing MM Therapy

The following decision-making stages for personalizing MM therapy are proposed:

(1) Pre-stratification

Identifying the indication (pain, MS-spasticity, or epilepsy), the pain/symptom phenotype, the patient’s goals, and the preferred route of administration. Consideration of risk factors (history of psychotic disorders, cardiovascular disease, advanced age, or pregnancy/breastfeeding) [16].

(2) DDI and PGx Risk Assessment

Identification of drugs with a narrow therapeutic index (e.g., clobazam, warfarin) and potential pharmacogenetic testing of the *CYP2C9/CYP2C19/CYP3A* pathways where the risk/uncertainty is high; planning biochemical monitoring (ALT/AST with CBD plus valproate) [2].

(3) Selection of the preparation and route of administration

Preference for standardized formulations with known THC and CBD content; consideration of oromucosal/oral preparations (greater predictability than inhalation) and lower THC doses in patients sensitive to central effects [77].

(4) Titration and Result Monitoring

“Start low, go slow,” assessing response after 2–4 and 6–8 weeks based on scales (e.g., NRS, BPI, spasticity NRS) and function, e.g., sleep; lack of response would indicate modification of the THC/CBD ratio or discontinuation [16].

(5) “Safety first”

Active monitoring for adverse effects (dizziness, drowsiness, or cognitive impairment), education on driving and operating machinery, liver enzyme monitoring with CBD, and medication review with each dose change [103].

In summary, this chapter concludes that the personalization of MM therapy should combine three layers: (i) clinical evidence (strongest for CBD in selected epileptic syndromes; moderate for MS spasticity; varied and dynamically growing for chronic pain); (ii) biology and pharmacology (PK/PD differences between routes of administration, involvement of CYP2C9/2C19/3A, and potential sex differences); and (iii) safety and interactions (especially CBD–clobazam and CBD–valproate). This allows for a rational, step-by-step adjustment of the composition (THC/CBD), route of administration, and dosage to the patient’s characteristics, with a realistic assessment of benefits and risks and a willingness to discontinue therapy if there is no response. Recent RCTs (e.g., *Nature Medicine* on chronic lower back pain) and the growing resources of RWE could improve personalization algorithms in the coming years, but adaptive and N-of-1 studies are still needed to more precisely identify responders and optimal dosage ranges [18].

## 8. Conclusions

Personalizing medical marijuana (MM) therapy requires simultaneous consideration of pharmacogenomics (PGx) and drug–drug interactions (DDIs), with dynamic phenotype adjustment (phenoconversion) depending on the current pharmacotherapy. In clinical practice, phenoconversion, i.e., changes in enzyme activity caused by concomitant pharmacotherapy or clinical conditions that can negate or reverse genotype-based predictions, should be considered. Therefore, DDGI assessment should become standard practice, with real-time phenotype correction within a CDS. This is illustrated, among other things, by the inhibition of CYP2C19 by CBD and the resulting interactions with clobazam, which highlights the need to interpret PGx results in the context of the current medication list and active monitoring of DDIs. In this context, the strongest and most well-established pillars are as follows: (i) *CYP2C9*–THC (variants *2/*3 result in slower clearance, higher exposure, and an increased risk of central effects); (ii) CBD–CYP2C19/3A–clobazam (enzyme inhibition, accumulation of *N*-desmethylclobazam, and the need for titration and monitoring); (iii) phenoconversion, which can modify genotype-based predictions. Integrating these layers into CDS systems (with real-time phenotype correction) should become the implementation standard.

In a clinical setting, the scope of “implementation maturity” includes safety, exposure (*CYP2C9* for THC; CBD–*CYP2C19* with clobazam), and the modification of neuropsychiatric risk by *AKT1*. At the same time, *COMT* Val158Met does not meet the criteria for a marker for routine use (lack of a consistent effect in meta-analysis). Preliminarily, promising multigene signals for analgesia (*ABCB1/TRPV1/UGT2B7*) require replication in prospective studies. In this review, we deliberately distinguish between (i) pathways where evidence supports immediate clinical implementation (*CYP2C9*–THC, CBD–*CYP2C19*/clobazam, and *AKT1*) and (ii) exploratory findings (multigenic analgesic signals and epigenetic signatures), which should currently be confined to research settings.

Epigenetics provides complementary insights. In humans, persistent methylation signatures associated with cannabis use are observed in the blood, as well as a partial reversibility of changes in sperm after a period of abstinence (~1 spermatogenesis cycle). The associations between cannabis use and epigenetic age (GrimAge) suggest potential cardiometabolic implications, but require cautious interpretation and control for co-exposures. Currently, epigenetic biomarkers are more of a perspective for exposure monitoring and safety than a tool for dose or chemotype selection.

The strength of the evidence varies between indications: For chronic pain, the analgesic effects are, on average, small to moderate, although more recent RCTs for a specific, standardized extract show clinically significant improvement. For spasticity in MS, the evidence is moderate (including a THC/CBD oromucosal spray). For developmental epilepsies, the efficacy of purified CBD is well-established and requires close monitoring of interactions (clobazam and valproic acid). From a personalization perspective, this means careful titration, a preference for standardized preparations, and active DDI/PGx monitoring.

Limitations of the evidence include heterogeneity of study designs and populations, lack of randomization in many RWE analyses, risk of confounding (co-exposures), and variability in product composition. In epigenetics, better mapping of target tissues and distinguishing between the reversible and permanent components over time and within developmental windows is crucial.

Practical implications include the following: (i) “start low, go slow,” with lower THC doses in CYP2C9*3 carriers and intensive education regarding sedation; (ii) with CBD and clobazam, slower titration, consideration of lower clobazam doses, and/or monitoring of *N*-desmethylclobazam, especially in *CYP2C19* IM/PM; (iii) integration of structured PGx results, DDI logic, and automatic phenotype correction into the EHR in CDS; (iv) preference for standardized preparations and routes of administration with predictable PK.

Among the research priorities are following: prospective RCTs and pragmatic studies comparing integrated strategies (PGx and DDI and phenotyping) with routine DDI controls; adaptive/N-of-1 studies to identify responders and optimize dose ranges and THC/CBD ratios; validation and calibration of multigene response predictors; and the development of a framework for epigenetic biomarkers (exposure/safety monitoring and potentially response prediction after rigorous validation).

In light of the current data, the greatest marginal benefits come from integration, meaning preventive PGx, ongoing DDI monitoring, and dynamic phenotype adjustment, supported by careful titration and safety monitoring, along with the development and validation of epigenetic biomarkers for clinical applications.

## Figures and Tables

**Table 1:** Phase I gene variants (CYP2C9, CYP2C19, and CYP3A4/5) and predicted metabolic phenotypes with PK/PD effects for THC/CBD.

Gene	Variant/Genotype	Predicted Metabolic Phenotype	Possible PK/PD Effect (THC/CBD)	Notes	Ref.
*CYP2C9*	*1/*1 (NM)	Normal metabolizer	Typical exposure to THC and 11-OH-THC; standard clinical response	Strong contribution of CYP2C9 to THC clearance shown in vitro and in silico	[2,3,4,5]
*CYP2C9*	*1/*2 or *1/*3 (IM)	Intermediate metabolizer (↓ activity)	↑ AUC/Cmax of THC and 11-OH-THC; potential increase in adverse effects (sedation, dizziness)	Effects depend on dose and route; consider careful dose titration	[2,3,4,5]
*CYP2C9*	*2/*2, *2/*3 or *3/*3	Poor metabolizer (marked ↓ activity)	Marked ↑ exposure to THC; higher risk of excessive psychoactive effects	Strongest effect with *3/*3; population differences in allele frequencies	[2,3,4,5]
*CYP2C19*	*1/*1 (NM)	Normal metabolizer	Typical rate of 7-OH-CBD (active metabolite) formation	CYP2C19 is key for CBD	[2,4,5]
*CYP2C19*	*2 or *3 (LOF); genotypes *1/*2, *1/*3, *2/*2, *2/*3, *3/*3	Intermediate/poor metabolizer	↓ Formation of 7-OH-CBD; potentially reduced metabolic activation	Impact on total CBD exposure may be complex (other compensatory pathways)	[2,4,5,6,8]
*CYP2C19*	*17 (GOF); genotypes *1/*17, *17/*17	Rapid/ultrarapid metabolizer	↑ Formation of 7-OH-CBD; possible ↓ exposure to parent CBD	Clinical relevance depends on indication and dose (e.g., epilepsy)	[2,4,5]
*CYP3A4*	Functional variants (e.g., *22)	Variable (often small) impact	Potential ↓ oxidative clearance of THC (8-OH-THC) and CBD; usually secondary effect	Contribution of CYP3A4/5 is smaller than that of CYP2C9 (THC) and CYP2C19 (CBD)	[2,3,4,5]
*CYP3A5*	*3/*3 (non-expresser) vs. *1 carrier	Absence/presence of enzyme expression	Minor differences in THC/CBD oxidation; clinical significance unclear	Role of CYP3A5 in cannabinoid metabolism appears limited	[2,3,4,5]

Arrow explanation: ↑—increase, ↓—decrease

**Table 2:** PGx–DDI–phenotype: THC/CBD—practical actions.

Pathway/Gene	Phenotype/Allele	DDI (Direction)	PK/PD Effect	Recommendation
*CYP2C9*	IM/PM: *2/*3 (and *2/*2, *3/*3)	― (no specific THC “perpetrator” DDIs; risk of phenoconversion with strong CYP2C9 inhibitors)	↑ AUC of THC and 11-OH-THC (especially after oral dosing) → ↑ risk of sedation/CNS AEs	Lower THC starting dose; slower titration (“start low, go slow”); educate about sedation/driving
*CYP2C19*	IM/PM (e.g., *2/*2, *2/*3, *2/*17)	CBD—inhibitor of CYP2C19/3A → ↑ *N*-desmethylclobazam (N-CLB) when combined with clobazam	↑ N-CLB exposure → ↑ risk of sedation/AEs; possible ↑ AUC of 7-OH-CBD	Consider reducing clobazam dose; slower CBD titration; monitor for sedation and/or N-CLB, especially in IM/PM
*UGT2B7*	― (polymorphisms of unclear effect; cannabinoid/drug influence is key)	CBD (more than THC) inhibits selected UGTs (UGT1A9/UGT2B7) → potential DDIs with glucuronidated drugs (e.g., opioids)	Possible ↓ clearance of UGT substrates → accumulation and AEs (e.g., respiratory depression with opioids)	Avoid high CBD doses with narrow-therapeutic-index glucuronidated drugs; consider clinical/lab monitoring
*ABCB1 (P-gp)*	rs2235048 and other transporter variants	― (not a DDI, but altered CNS transport)	Differences in acute psychoactive response (e.g., after inhalation) due to blood–brain barrier transport of cannabinoids	Caution with high-THC products; patient education; consider avoiding highly psychoactive chemotypes in sensitive individuals

Arrow explanation: ↑—increase; ↓—decrease; →—leads to/results in.

**Table 3:** Cannabis-related DNA methylation signatures and reversibility across tissues.

CpG Markers/Signature	Direction of Change in Users vs. Non-Users	Typical Δβ Range	Tissue/Sample Type	Reversible? *
EWAS panel of cannabis-associated CpGs (CARDIA; ~201 sites)	Mixed (both hyper- and hypomethylation)	Usually Δβ < 5%	Peripheral blood (adult cohorts)	Partly reversible vs. exposure; some sites track recent use, others reflect cumulative exposure and persist in former users
Trans-ancestry EWAS meta-analysis (ever vs. never use)	Mixed; small effect sizes at individual CpGs	Usually Δβ < 5%	Peripheral blood (multi-cohort blood samples)	Likely stable exposure markers; persistence observed in midlife long-term users
Sperm CpGs in male cannabis/THC users	Both hyper- and hypomethylation at thousands of CpGs, enriched in developmental genes	Small-to-moderate changes at many sites (individual loci with larger Δβ)	Sperm (human; supported by rat THC models)	Partly reversible after ~11 weeks of abstinence (≈one spermatogenesis cycle), but a subset of loci remains differentially methylated
CpGs in placenta/fetal tissues after prenatal THC exposure	Mixed; enrichment in neurodevelopmental pathways	Small-to-moderate Δβ (locus-dependent)	Placenta and fetal/neurodevelopmental tissues	Not clearly reversible; signatures appear persistent across development in animal/human translational data 3

* Reversibility column is based on longitudinal/abstinence data where available and on cross-sectional persistence in former users.

**Table 4:** Implementation maturity of PGx/epigenetic signals in MM personalization.

Pathway/Gene	Phenotype	Evidence Level	Clinical Readiness
*CYP2C9–THC*	Exposure/sedation	RCT + PK studies	Ready for dose-titration recommendations
*CBD–CYP2C19–clobazam*	N-CLB accumulation, AEs	RCT + PK	Ready for monitoring/dose adjustment
*AKT1 rs2494732*	Acute psychotomimetic effects, psychosis risk	Translational + human cohorts	Useful for high-risk stratification, not routine
*COMT Val158Met*	Cannabis × psychosis	Meta-analysis negative	Not recommended for routine use
*ABCB1/TRPV1/UGT2B7*	Analgesic response	Single observational cohort	Exploratory only
Epigenetic signatures	Exposure, epigenetic age	EWAS, sperm studies	Safety/exposure research tools

## Data Availability

No new data were created or analyzed in this study. Data sharing is not applicable to this article.

## Abbreviations

11-OH-THC—11-hydroxy-THC, the primary oxidative metabolite of THC; 2-AG—2-arachidonoylglycerol, an endogenous cannabinoid; *ABCB1* (P-gp)—ATP-binding cassette subfamily B member 1 (P-glycoprotein transporter); AEA—anandamide, an endogenous cannabinoid; *AKT1*—AKT serine/threonine kinase 1, a modifier of the acute psychotomimetic response to THC; AUC—area under the concentration–time curve (drug exposure); BPI—Brief Pain Inventory; CB1/CB2—cannabinoid receptors type 1 and 2; CBD—cannabidiol; CDS—Clinical Decision Support; *CNR1/CNR2*—genes encoding CB1/CB2 receptors; COMT—catechol-O-methyltransferase; *COMT* × cannabis interaction not consistently supported; CpG—cytosine–phosphate–guanine dinucleotide sites (DNA methylation); CPIC—Clinical Pharmacogenetics Implementation Consortium; *CYP2C19*—cytochrome P450 2C19 (phase I metabolism; key for CBD 7-hydroxylation); *CYP2C9*—cytochrome P450 2C9 (phase I metabolism; major for THC oxidation); DDI—drug–drug interaction; DDGI—drug–drug–gene interaction; DNAm—DNA methylation; DNMT/TET—DNA methyltransferases/ten-eleven translocation enzymes; *DPYD*—dihydropyrimidine dehydrogenase gene (PGx example); ECS—endocannabinoid system; EHR—electronic health record; EWAS—epigenome-wide association studies; FAAH—fatty acid amide hydrolase (ECS enzyme); GOF/LOF—gain-of-function/loss-of-function variants; GPCR—G-protein-coupled receptor; GrimAge—epigenetic age estimator used in exposure studies; H3K4me3/H3K27me3—histone marks (bivalent chromatin landscape); HAT/HDAC—histone acetyltransferases/histone deacetylases; IM/PM/UM—intermediate/poor/ultrarapid metabolizer phenotypes; INR—international normalized ratio (anticoagulation monitoring); lncRNA/miRNA—long/micro non-coding RNA; MM—medical marijuana (medical cannabis); N-CLB—*N*-desmethylclobazam, active clobazam metabolite; NRS—Numerical Rating Scale (pain/spasticity); PBMC—peripheral blood mononuclear cells; PCI—percutaneous coronary intervention (PGx use case outside cannabis); P-gp—P-glycoprotein transporter (product of ABCB1); PK/PD—pharmacokinetics /pharmacodynamics; PGx—pharmacogenomics; RCT—randomized controlled trial; RRBS/WGBS—reduced-representation/whole-genome bisulfite sequencing; RWE—real-world evidence; *SLCO1B1*—gene encoding OATP1B1 transporter (PGx example); THC—Δ^9^-tetrahydrocannabinol; THC-COOH—11-nor-9-carboxy-THC, acidic THC metabolite; *UGT1A1/UGT1A9/UGT2B7*—UDP-glucuronosyltransferases (phase II metabolism); 7-OH-CBD—7-hydroxy-CBD, active CBD metabolite.
